# The Localization of Long-Distance Dependency Components: Integrating the Focal-lesion and Neuroimaging Record

**DOI:** 10.3389/fpsyg.2016.01434

**Published:** 2016-09-30

**Authors:** Maria M. Piñango, Emily Finn, Cheryl Lacadie, R. Todd Constable

**Affiliations:** ^1^Language and Brain Lab, Department of Linguistics, Yale UniversityNew Haven, CT, USA; ^2^Interdepartmental Neuroscience Program, Magnetic Resonance Research Center, Yale UniversityNew Haven, CT, USA

**Keywords:** left inferior frontal cortex, Broca's and Wernicke's aphasia, supplementary motor area, precuneus, long-distance dependencies, sentence comprehension, working memory, attention

## Abstract

In the sentence “The captain who the sailor greeted is tall,” the connection between the relative pronoun and the object position of *greeted* represents a long-distance dependency (LDD), necessary for the interpretation of “the captain” as the individual being greeted. Whereas the lesion-based record shows preferential involvement of only the left inferior frontal (LIF) cortex, associated with Broca's aphasia, during real-time comprehension of LDDs, the neuroimaging record shows *additional* involvement of the left posterior superior temporal (LPST) and lower parietal cortices, which are associated with Wernicke's aphasia. We test the hypothesis that this localization incongruence emerges from an interaction of memory and linguistic constraints involved in the real-time implementation of these dependencies and which had not been previously isolated. Capitalizing on a long-standing psycholinguistic understanding of LDDs as the workings of an active filler, we distinguish two linguistically defined mechanisms: *GAP-search*, triggered by the retrieval of the relative pronoun, and *GAP-completion*, triggered by the retrieval of the embedded verb. Each mechanism is hypothesized to have distinct memory demands and given their distinct linguistic import, potentially distinct brain correlates. Using fMRI, we isolate the two mechanisms by analyzing their relevant sentential segments as separate events. We manipulate LDD-presence/absence and *GAP-search* type (direct/indirect) reflecting the absence/presence of intervening islands. Results show a *direct GAP-search*—LIF cortex correlation that crucially excludes the LPST cortex. Notably, *indirect GAP-search* recruitment is confined to supplementary-motor and lower-parietal cortex indicating that *GAP* presence alone is not enough to engage predictive functions in the LIF cortex. Finally, *GAP-completion* shows recruitment implicating *the dorsal pathway* including: the supplementary motor cortex, left supramarginal cortex, precuneus, and anterior/dorsal cingulate. Altogether, the results are consistent with previous findings connecting *GAP-search*, as we define it, to the LIF cortex. They are not consistent with an involvement of the LPST cortex in any of the two mechanisms, and therefore support the view that the LPST cortex is not crucial to LDD implementation. Finally, results support neurocognitive architectures that involve the dorsal pathway in LDD resolution and that distinguish the memory commitments of the LIF cortex as sensitive to specific language-dependent constraints beyond phrase-structure building considerations.

## 1. Introduction

A long-distance or filler-gap dependency (LDD) is a syntactico-semantic relation between a pronominal element and a syntactically licensed position, or GAP, in an embedded clause. The LDD is thus the linguistic device that allows the pronominal element to be interpreted within the embedded clause. In the English sentence “*The captain*_*k*_ *[who*_*k*/*j*_ *the sailor predicted that the weather would frighten (GAP)*_*j*_*] smiled.*” the LDD is the connection between the relative pronoun and the object position of *frighten*, to which the semantic role of *frightenee* is assigned. LDDs have traditionally provided a window to explore the interaction between lexico-semantic and syntactic mechanisms involved in sentence composition, and have thus represented a rich space for neurolinguistic and psycholinguistic investigation. In LDDs, these mechanisms are specifically observed in the interpretation of the relative pronoun both as the object of the embedded verb (e.g., *the frightenee*) and as the coreferent to the head noun antecedent (e.g., *The captain*), mechanisms that are presumably grounded not only in fundamental properties of sentence composition such as argument structure licensing and discourse linking but also in the neurological properties of the linguistic subsystems that support those properties (e.g., Frazier et al., 1983; Frazier and Clifton, 1989; Grodzinsky, 1989; Swinney et al., 1989; Swinney and Zurif, 1995; Gibson, 1998; Grodzinsky, 2000; Phillips, 2003; Avrutin, 2006).

From a neurolinguistic perspective, LDD implementation also allows us to investigate how the interaction between sentence composition and memory should be understood, as well as what the cortical distribution of this interaction should be. Interpretation of the relative pronoun is, after all, expected to place significant demands on the memory system: the pronoun must be held in memory while the intervening syntactic and semantic material is parsed (in the present case “*that the weather would*”). The presence of intervening material taxes the processing system (e.g., King and Kutas, 1995; Cooke et al., 2002; Fiebach et al., 2002; Santi and Grodzinsky, 2012; Santi et al., 2015) and is subject to aging effects (Zurif et al., 1995). So, understanding the cortical distribution of these dependencies gives us insight into the basic commitments that any neurocognitive model of language must allow with respect to sentence composition in addition to the interactions of sentence composition with other components of cognition, most notably memory.

The record on LDD comprehension reveals a long-standing incongruence regarding the language processing commitments of the left inferior frontal (LIF) cortex: lesion studies show that in contrast to Wernicke's patients and patients with lesions in the right hemisphere homolog of Broca's area, Broca's patients fail to implement LDDs in a normal fashion during real-time comprehension. Specifically, these subjects fail to show normal implementation of the “GAP-filling” effect: the reactivation of the antecedent (i.e., the entity coreferent with the relative pronoun) at the position of the GAP (e.g., Zurif et al., 1993; Swinney et al., 1996; Grodzinsky et al., 1999; Grodzinsky, 2000; Burkhardt et al., 2003; Love et al., 2008). Given the localization value of Broca's and Wernicke's aphasia, this pattern of performance is taken to indicate that LDDs demand the workings of the LIF cortex and, crucially, do not depend on the workings of the left posterior superior temporal (LPST) cortex. By contrast, neuroimaging work has shown equal engagement of the LIF cortex *and* the LPST cortex for the implementation of the same dependencies (e.g., Stromswold et al., 1996; Cooke et al., 2002; Fiebach et al., 2002; Ben-Shachar et al., 2003, 2004; Friederici et al., 2003; Grodzinsky and Friederici, 2006; Santi and Grodzinsky, 2008).

We take both sets of results– lesion- and neuroimaging-based– to be valid and on that basis propose that together they provide complementary observations about LDDs and the neurocognitive resources that support them. Specifically, we hypothesize that one crucial property of LDD implementation–*GAP-search*–relies on the workings of the LIF cortex, as the lesion-based record shows. This leaves open the question of the role of the LPST cortex reported in the neuroimaging record. In this respect we test the hypothesis that such LPST cortical recruitment would not be connectable to the implementation of *GAP-search*; and may be instead implicated in *GAP-completion*, a local, lexically-driven process fundamental to all sentence composition. To this end, we isolate the neurocognitive factors underpinning LDD comprehension on the basis of an analysis of relative pronouns that connects to parallel, incremental left-to-right structure-building mechanisms with potential neurocognitive relevance. Using fMRI, we examine the timing and cortical commitments of the interaction of these mechanisms. We conclude with a discussion of the implications of these findings for the lesion vs. imaging “mismatch,” and in the context of current neurocognitive models for our understanding of the LIF cortex as a “language” area.

### 1.1. The structural and processing properties of long-distance dependencies

The purpose of this section is to present the linguistic structure for long-distance dependencies (LDDs) that supports their real-time processing implementation. This structure is therefore the basis for the definitions of the processing mechanisms of *GAP-search* and *GAP-completion*, which operationalize the dependency in neurocognitive terms^1^. In English, long-distance dependencies prototypically emerge in relative clause and *wh*-question formation. In the case of relative clauses, they involve three main elements: the antecedent, the relative pronoun, and the GAP. The antecedent is the denotation of the head noun of the noun phrase containing the relative clause [*captain* in (1) below]. The RELPRO (which may be phonologically empty in English) is the entity that semantically links the antecedent and the GAP [*who* in (1) below]. The RELPRO occupies what we would call a “non-canonical” position, a position that does not receive direct semantic role assignment by a predicate, and therefore does not receive direct interpretation with respect to the proposition associated with the embedded clause. This interpretation is provided instead through the dependency it forms with the GAP. The GAP, in turn, is a hypothesized phonologically empty syntactically valid place-holder of the “displaced” relative pronoun which receives a semantic role by virtue of its grammatical function within the embedded clause. (1) below illustrates the relation between the GAP to which the semantic role of “experiencer” is assigned and the denotation of the head noun *captain* (the antecedent):

(1) The captain_*antecedent*_ [who the sailor predicted that the weather would frighten (“the captain”)_*GAP*_] turned back to port.

The relation between the antecedent and the GAP is mediated by the relative pronoun (RELPRO). The RELPRO holds a coreference relation with the antecedent. And it is this coreference relation between the RELPRO and the antecedent that allows the antecedent to be interpreted as a participant in the proposition associated with the embedded clause, i.e., *the sailor predicted that the weather would frighten* ***the captain***. Establishing an LDD therefore means connecting, on the one hand, the antecedent and the RELPRO and, on the other, the RELPRO and the GAP. These two distinct links are identified by the (shared) indices in (2) below:

(2) The captain_*k*_ [who_*k*/*j*_ the sailor predicted that [the weather would frighten (GAP)_*j*_]] turned back to port.

As can be seen, LDDs contain syntactic (construal of the relative pronoun as a grammatical relation in a“noncanonical” position) and lexico-semantic (semantic role assignment) mechanisms which are categorically distinct, and consequently subject to at least partially independent principles of composition. They also involve pronoun interpretation (the establishment of coreference between the RELPRO and the antecedent), which, at least for processing purposes, is identified as a discourse process (e.g., Grodzinsky et al., 1991; Avrutin, 1999; Piñango and Burkhardt, 2005). We take these mechanisms to be encoded in the lexical representation of the RELPRO itself as syntactic, discourse, and semantic selectional requirements respectively. The proposed representation is presented in (3) below:

**Table:** 

(3) RELPRO “who”
	{Syn:	[*_NP_* N*_head_*	[*_CP_* **NP**_*k*/*j*/*w*_	[*_IP_* NP*_j_*	[V NP*_w_*]]]]}
	{Sem:	[argument*_k_*	[**pronoun**_*k*/*j*/*w*_	[predicate	[argument*_j_*, argument*_w_*]]]]}
	{Discourse:	[antecedent*_k_*	[ **PRO**_*k*/*j*/*w*_	[SUBJ*_j_*	[OBJ*_w_*]]]]}
	{Phon:	[hu] }			

The representation in (3) specifies the syntactic, discourse, and lexico-semantic environments in which the RELPRO *who* may be licensed, thus capturing the main properties of its linguistic distribution in English. Retrieval of a RELPRO during comprehension therefore means the retrieval of this lexical *composite* with all the mutually constraining algorithms that determine the environment of its realization. In this way the lexical entry itself makes explicit the possible predictions by the parser regarding preceding and crucially, incoming lexical material.

This description thus represents the relevant lexico-syntactic characterization that we take to underlie both the filler-gap effect (e.g., Crain and Fodor, 1985; Stowe, 1986; Swinney et al., 1988; Frazier and Flores d'Arcais, 1989; MacDonald, 1989; McElree and Bever, 1989; Nicol and Swinney, 1989; Fodor, 1995) and its corresponding psycholinguistic generalization, the *Active Filler Hypothesis* (Frazier and Clifton, 1989). Specifically, in this linguistic articulation, the GAP is simply the realization of a coindexation relation between the relative pronoun and a phonologically unsupported [NP+semantic argument+grammatical relation] “triplet” in the embedded IP. The Active Filler Strategy therefore emerges as the implementation of the search to satisfy the RELPRO's requirements^2^. We conjecture that the explicitness of this lexically “packaged” parallel, multi-layer structure is what gives the LDD its seemingly unified processing implementation, what informs the parser as to the syntactic constituents where it can/cannot find a GAP (e.g., Stowe, 1986), and what so powerfully drives the RELPRO (the filler) to hypothesize a GAP even in constructions where it will ultimately be disallowed (e.g., Frazier et al., 1983; Hickok, 1993).

Having made explicit the necessary linguistic and psycholinguistic considerations, we turn to other non-linguistic real-time implementation requirements, specifically, memory requirements. We observe that there are in principle three “inflection points” in the LDD processing: the signaling by RELPRO retrieval that a GAP is incoming, the search for the GAP, and the actual instantiation of the GAP; that is, the point in the composition of the embedded clause where the RELPRO requirements are met (i.e., the GAP). We reason that whereas the antecedent-RELPRO coreference relation and GAP instantiation are unambiguous and local, the instantiation of the search for the GAP is, by contrast, multiply ambiguous due to the availability of multiple potential GAP positions that the RELPRO can be coindexed with and that are associated with all the possible grammatical relations in the embedded clause. This inherent ambiguity is presumably what forces the processor to closely track the syntactic and semantic structure of the incoming embedded clause until the GAP is reached, thus making it memory taxing. It is this basic difference what makes the gap search process a clearer candidate for the probing of cortically localizable real-time linguistic processes.

On this basis, we articulate the LDD into two linguistically distinct stages, the search process itself vs. the licensing point of the GAP. These stages are in turn operationalizable as two mechanisms distinguishable by their differing memory demands. Those mechanisms are:

- *GAP-search*: triggered by the retrieval of the RELPRO. It is the language composition process where memory resources are maximally taxed: Upon retrieval of the RELPRO its lexico-syntactic requirements must be satisfied all while the phrase structure and semantic representations of the embedded-clause are being composed [e.g., *who*_*k*/*j*_ *the sailor predicted that the weather*, in (2)]. *GAP-search* is the mechanism that effectively implements the Active Filler Strategy: the RELPRO's lexically-driven search within the embedded clause in order to meet its lexico-syntactic requirements.
- *GAP-completion*: triggered by the retrieval of the embedded verbal predicate. It is the process whereby the RELPRO's lexico-syntactic requirements are satisfied. In (2) this process takes place when the embedded verb is retrieved: the earliest point at which the embedded predicate (e.g., *frighten*) can license the object grammatical relation/NP structure and assign to the RELPRO the corresponding semantic role (e.g., *frighten-ee*). This not only completes the interpretation of the RELPRO *who* within the embedded clause but “grounds it,” as it were, into the composition of the rest of the embedded clause. It allows the interpretation of the coreferring antecedent (the denotation of the matrix subject head, *captain*) as a participant in the embedded proposition's semantic representation. Crucially, this process, like *GAP-search*, is compositional and therefore expected to require memory resources beyond lexical retrieval. However, given the locality of its resolution, the amount of memory resources *GAP-completion* demands should be significantly less than those demanded by *GAP-search*.

Here, we hypothesize that given their respective linguistic properties and correlated memory demands, these two mechanisms are potentially neurologically dissociable in a way that could shed light on the neurocognitive incongruence at issue. Notably, this kind of processing analysis finds direct support in previous findings by Phillips et al. (2005). That report presents two distinct electrophysiological components associated with long-distance dependency comprehension: a sustained anterior negativity subsequent to the initiation of the *wh*-dependency and a late posterior positivity (P600) associated with the completion of the dependency. We take that pattern to represent the electrophysiological correlates of *GAP-search* and *GAP-completion* respectively and thus take them as initial support for the analytical approach adopted here.

Most crucially for our present purposes however, a closer look at the fMRI record also suggests the potential viability of this dissociation. We turn to that record directly below.

### 1.2. LDDs and the LIF cortex in fMRI: previous experimental record

In this section we discuss previous neuroimaging work that has also targeted either *GAP-search* or *GAP-completion* as we define them here in connection to the workings of the LIFG. Our search through the record was constrained by the requirement that the given report target one, the other, or both mechanisms in question as unified phenomena. The conclusions from that work together with the lesion-based evidence constitute the basis for the specific localizational predictions that we test^3^. Of the large body of neuroimaging work on LDD comprehension, four reports specifically deal with *GAP-search* as we have defined it: (Santi and Grodzinsky, 2007, 2010, 2012) and Matchin et al. (2014). Interestingly, we found no previous work on LDD comprehension targeting *GAP-completion*. In line with the focal lesion evidence these four reports converge on the observation that at least *GAP-search*, as we have defined it here, preferentially recruits the workings of the the LIF cortex. This is what unites them. In what follows we discuss for each of the reports the specifics of how these observations came to be.

Santi and Grodzinsky (2007) connect LDDs to the LIF cortex exclusively through what they call a “distance” effect. They test two phenomena. The one at issue involves object relatives in three conditions: one-NP embedded subject, two-NP embedded subject, and three-NP embedded subject. Crucially, these added NPs are irrelevant to the structure of the RELPRO-GAP dependency itself as the NPs have been added to the embedded subject phrase. Their function in the experimental design is to add material (specifically NP material which is syntactically identical to the RELPRO) between the RELPRO and the object-GAP. This material does not add to the complexity of the LDD but does increase the linear distance between the RELPRO and the GAP. In so doing, it increases the amount of structure the parser must build in order to get to the GAP. Such increase is coupled with an increase in number of nominals (one to three). Santi and Grodzinsky (2007)'s results show recruitment of the LIF cortex in the three vs. two nominal increment. We see this manipulation as addressing *GAP-search* as we have defined it (to the exclusion of *GAP-completion*) because in the three-NP condition, the minimal difference was the increase in distance between the RELPRO and the GAP, and this greater distance had to be tracked in order for the parser to get to the GAP^4^.

More recently, Santi and Grodzinsky report in two separate papers, 2010 and 2012, an association between the LIF cortex and LDD processing which, given their respective designs, again target *GAP-search* to the exclusion of *GAP-completion*. Whereas in Santi and Grodzinsky (2010) the manipulation involves a comparison between *GAP-search* and embedding, connecting only *GAP-search* to the LIF cortex, Santi and Grodzinsky (2012) distinguishes general *dependency* from *predictability*, the ability of the parser to predict the need for a GAP. Their results show that *predictability* not *dependency* correlates with the LIF cortex effect, focused on BA 45^5^.

Finally, Matchin et al. (2014) test the hypothesis that the LIF cortex supports a more general “antecedent-variable” dependency function, thus allowing the possibility to consider *GAP-search* as a member of a larger family of “search”-based processes. Such a hypothesis predicts an LIF cortex preferential activation for *pronoun-antecedent* relations (i.e., backward anaphora) which, like RELPRO-based LDDs, contain as a “variable” an element with an incomplete referential interpretation (pronoun) which must actively look for an “antecedent,” the entity with which it must corefer. As with Santi and Grodzinsky (2007), the experimental design of Matchin et al. (2014) targets the *GAP-search* portion of the pronoun-dependency, as we have defined it. Their results show that only the subtractions involving backward anaphora (and not the RELPRO-based LDDs) yielded LIF cortex activation. And for these there was, in addition, activation in the right MTG, STC, bilateral SMA, bilateral occipital activation, and left STS. So, even though the observation is clearly made that the LIF cortex participates in predictive searches similar to *GAP-search*, it is also the case that other cortical regions also participate in this process, rendering the specific contribution of the LIF cortex in the processing of this kind of LDD inconclusive. This said, the presence of LIF cortex activation in this fairly different kind of dependency is suggestive of a deeper processing commonality, which so far has not been fully explored in the neuroimaging literature, and is one that we think may be captured by the generality of the *GAP-search* mechanism^6^.

In sum, whereas the vast majority of fMRI research involving LDDs correlate them to cortical regions beyond the LIF cortex, some do provide exclusive or close to exclusive correlation with LIF cortex. Those that do, target *GAP-search* as we have defined it. By contrast, *GAP-completion*, the other major LDD mechanism capturing the more general properties of LDD composition, remains less explored. In light of this, and in order to further understand the factors involved in the neurocognition of LDDs we ask the following questions: What is the neurocognitive relation between *GAP-search* and *GAP-completion*? Do they rely on the workings of overlapping brain regions? And, could we associate *GAP-completion* to the LPST cortex, thus directly addressing the lesion-neuroimaging incongruence? In addition, a new question is revealed: if the effects reported reflect *GAP-search*, why are they observed mainly in the context of *object*-relative GAPs? The specifics of the study seeking to address these question are presented directly below.

### 1.3. The study: determining the neurological underpinnings of LDDs

Our analysis above shows that LDD comprehension can be organized into at least two processing mechanisms. We propose here that the existence of this dual mechanism infrastructure and the differential memory resources that it demands is the source of the disparity regarding the cortical recruitment of LDD processing. Moreover, we propose that the reason it has not been detected before has been due to a limitation inherent to the traditional data-analysis approach used in the past. We thus propose that the cortical localizational incongruence is the result of the interaction of two factors: one linguistic and one methodological. The **linguistic factor** refers to the previous analyses which collapse *GAP-search* with RELPRO interpretation at the GAP position, *GAP-completion*, thus conflating processes with potentially distinct neurocognitive demands. The **methodological factor** refers to the traditional approach to data analysis in language-related fMRI whereby subtractions take place at the *sentence level*, an approach which, in this case, prevents finer-grained exploration of the intra-sentential components of the dependency.

We address the linguistic factor by testing constructions that vary the degrees of linguistic compositional demands and in doing so allow us to examine the two mechanisms separately. These compositional demands range from a condition where an LDD is not required, as in (4):

(4) The captain **believed** the sailor's prediction yesterday **that** the weather would frighten_*no*−*gap*_ **the crew** and turned back to port. (Condition D)

to one where an LDD is required and the link between the RELPRO and the GAP is syntactically direct, as in (2) above repeated here as (5):

(5) The captain_*k*_ [**who**_*k*/*j*_ the sailor predicted that the weather would frighten (GAP)_*j*_] turned back to port. (Condition A)

to one where the syntactic connection between the RELPRO and the GAP is not direct [i.e., the intervening syntactic constituent does not contain the predicate licensing the GAP (6)]^7^:

(6) The captain_*k*_ **[who**_*k*/*j*_ [the sailor's prediction yesterday about the weather] had frightened_*gap*_, turned back to port. (Condition B/C)

Comparing these conditions allows us to observe the extent to which the memory-language interaction is sensitive to actual compositional linguistic mechanisms, and if so, which ones and with what cortical implications. In this respect, (5) > (4) and (6) > (4) in particular allow us to assess the cortical resources that must be recruited as the processor actively searches for the GAP [(5) > (4)] vs. those which must be recruited during the composition of sentence structure which the processor “knows” cannot contain a GAP, as in [(6) > (4)] (see Stowe, 1986; Kluender, 1998, respectively, for early evidence of the sensitivity of the processor to island constraints, and of how, and in contrast to widespread assumptions in linguistics, islands could in fact result from the interaction of processing factors).

With these contrasts in place, we are able to discuss our approach to the examination of the role of memory in the long-distance dependency construction. We do this through a data analysis manipulation whereby the two hypothesized processing mechanisms, *GAP-search* and *GAP-completion* are analyzed as separate *events*. Specifically, we use an intra-sentential event-related subtraction approach whereby subtractions are performed over the relevant non-overlapping segments of the sentence (see Data Analysis section below for technical details). This, in combination with the minimal contrasts in the linguistic manipulation between conditions, presence/absence of GAP and presence/absence of direct antecedent-GAP link, allows us to isolate simple phrase-structure building from active *GAP-search* and from *GAP-completion*, respectively. The details of the experimental design and data analysis are presented directly below (see Lai et al., in press for a similar use of event-related design in the context of semantic composition).

## 2. The study: investigating *GAP-search* and *GAP-completion*

### 2.1. Materials

The study contained a total of four conditions (A, B, C, and D) with 60 sentences in each of the conditions. Sentences were constructed as matching quadruples, thus controlling for non-relevant lexico-semantic and syntactic factors. This resulted in a final script of 240 sentences (60 quadruples). Test sentences for Conditions A and B were directly modeled from Gibson and Warren (2004), which introduces the ± direct RELPRO-GAP link manipulation. A sample of a quadruple is presented in Table 1 below. As can be seen, whereas the conditions differ in the relevant syntactic properties (e.g., verbal vs. nominal: “sailor predicted” vs. “sailor's prediction”) they share all other main lexico-semantic components, thus ensuring that they were as close as possible in terms of number of words, word frequency, and sense co-occurrence. Given our interest in separating activation related to *GAP-search* from that related to *GAP-completion* our unit of analysis was the *Event* which was a segment of the sentence. Accordingly, condition matching had to be implemented especially at the event level. For matching (and data analysis) purposes then each sentence was construed in terms of three events which in Table 2 are observable in the internal bracketing of the sentences: **Event 0** contains the material before the brackets including head noun and relative pronoun/verb, **Event 1** corresponding to *GAP-search* contains the material in bold within brackets; and **Event 2** corresponding to *GAP-completion* contains the material after the brackets. As can be seen, for Event 1, all conditions match in terms of number of words. For Event 2, condition D, the control condition has in addition three words corresponding to the object NP (two words) and the conjunction (one word). We note that as this is the control condition any extra activation associated with the three extra words would be eliminated in the subtraction process. (For further description of the analysis approach see Table 3 in the Data Analysis section).

**Table 1:** **Four experimental conditions**.

	**Sentence**	**Condition**
A	The captain, who **[the sailor predicted yesterday that the weather]** would frighten_*gap*_,turned back to port.	*GAP-search*/direct and *GAP-completion*
B	The captain, who **[the sailor's prediction yesterday about the weather]** had frightened_*gap*_,turned back to port.	*GAP-search*/indirect and *GAP-completion*
C	^*^The captain, who **[the sailor's prediction yesterday about the weather]** had frightened the crew, turned back to port.	*GAP-search*/indirect and *GAP-completion* violation
D	The captain believed **[the sailor's prediction yesterday that the weather]** would frighten the crew and turned back to port.	No GAP-search and No GAP-completion

**Table 2:** **Experimental conditions by events**.

**Condition**	**Event 0**	**Event 1 (± *GAP-search*)**	**Event 2 (± *GAP-completion*)**
A	The politician who	the journalist claimed that the government report	had bothered_*gap*_ is calling a press conference
B	The politician who	[the journalist's claim about the government report]_*island*_	had bothered_*gap*_ is calling a press conference
C	The politician who	[the journalist's claim about the government report]_*island*_	had bothered the people is calling a press conference
D	The politician believed	the journalist's claim that the government report	had bothered the people and is calling a press conference

**Table 3:** **Planned subtractions by events: single subtractions**.

**Subtraction**	**Event 1**	**Event 2**
A > D	*GAP-search_direct_*	*GAP-completion*
B > D	*GAP-search_indirect_*	
A > B	*GAP-search* (*GAP-search_direct_* – *GAP-search_indirect_*)	–
B > C	–	*GAP-completion*

Table 1 presents the conditions with their respective dependencies. Figure S1 in the Supplementary materials presents the corresponding syntactic structures (Note that for Conditions A vs. B/C, the different syntactic structures determine the nature of the link between the RELPRO and GAP: *direct* for A and *indirect* for B). Asterisk (*) in Condition C signals ungrammaticality.

In addition, the A, B, and D conditions were pre-tested for acceptability using a five-point likert scale. This pre-test allowed us to ensure that even though D would be more acceptable than A and B, there would be no difference in acceptability between A and B conditions. And this is what planned comparisons show. As expected Condition D [D_*mean*_ = 3.79 (*SD* = 0.5)] was deemed significantly more acceptable than conditions A [A_*mean*_ = 2.66 (*SD* = 0.5) (*t* = −4.05, *p* < 0.001)] and B [B_*mean*_ = 2.67 (*SD* = 0.6) (*t* = −4.1, *p* < 0.001)]. Also as expected no statistical difference in acceptability between A and B was found (*t* = −0.03, *p* = 0.48). This was calculated on the basis of responses from a sample of 13 native English speakers from the Yale undergraduate population, the same population from which the fMRI participants were selected.

Comprehension questions followed all condition A, B, and D sentences. No questions followed condition C sentences as the kind of ungrammaticality in that condition makes it difficult to ask questions that have an unambiguous yes/no answer. This said, we note that the ungrammaticality in Condition C appears toward the end-of the sentence, crucially, at the *GAP-completion* segment sentence. So, subjects could not know during the first part of the sentence up to the embedded verb whether they were in the presence of a grammatical or ungrammatical sentence. This motivated them to pay attention to all sentences equally.

In addition, questions probed different combinations of the matrix subject, embedded subject, matrix verb, and embedded verb. This variability was introduced intentionally to motivate participants to pay attention throughout the sentence as opposed to specific features of the sentence. To further minimize strategizing, the assignment of a given question to a given sentence was random, so even if the participants could realize that the matrix/embedded subject nouns and the matrix/embedded verbs mattered, for any given sentence they could not predict what specific element would be queried. So, they had to pay attention to all components of the sentences equally. For a sentence like *The captain, who the sailor predicted yesterday that the weather would frighten, turned back toward port.*, subjects would get one of these possible questions:

- Did the sailor predict that the weather would frighten the captain? (expected answer: Y)
- Did the captain predict that the weather would frighten the sailor? (expected answer: N)
- Did the captain turn back toward port? (expected answer: Y)
- Did the sailor turn back toward port? (expected answer: N)

Coming back to the experimental sentences, this is what each condition probes:

**Condition A** examines *GAP-search*, triggered at *who* and *GAP-completion*. The distance between the RELPRO and the GAP is expected to reveal the workings of the memory system in a situation where finding the GAP is expected, given the absence of intervening islands, as compared to Condition D, the no-GAP condition, and Condition B, the island condition where the GAP is not expected *within* the local constituent.
**Condition B** also combines *GAP-search* (triggered at *who*) and *GAP-completion*. However, in contrast to Condition A, in Condition B the search for the GAP must bypass the embedded subject (which is an island). Bypassing the embedded subject means that the processor needs to wait for that NP constituent to end to find the GAP. That is what the B>D contrast is intended to reveal.

For both A>D and B>D contrasts there is a clear interaction with the memory system in connection to *GAP-search*. So similarity in recruitment is expected. A difference in recruitment (A>D) and (B>D) would then be interpreted as a difference in the *quality* of the interaction with respect to *GAP-search*, one where the processor is not actively looking for the GAP (B>D), vs. one where it is (A>D).

**Condition C** is identical to Condition B except that the GAP position has been filled with an additional NP, which renders the sentence ungrammatical. The motivation for this condition focuses on the possible distinct cortical recruitment associated with *GAP-completion*. If, as we hypothesize, *GAP-completion* has distinct neurological commitments from *GAP-search*, this process will be observed as a unique activation pattern when comparing Conditions B>C, as these two conditions differ only with respect to the *GAP-completion* factor. B>C thus effectively brings us the closest to observing the preferential recruitment for *GAP-completion* alone.
**Condition D** represents the control condition. It has the same number of words and constituents as the Condition A and B counterparts, thus equally requiring full phrase-structure building and semantic composition. It lacks a long-distance dependency, so it is expected to tax the memory system the least in comparison to Conditions A or B.

### 2.2. Design

Each subject was presented with the 240-sentence script containing the 4 conditions, A, B, C, and D (60 items per condition). No additional fillers were included in the script. All 240 sentences were distributed in a pseudo-random fashion in 10 separate runs of 24 sentences each. The four experimental conditions were distributed in a counterbalanced fashion within each run such that no two sentences of the same quadruple would be included in the same run. Each subject was presented with a unique order of runs. So, in the end no two subjects saw the exact same sentence presentation order.

Each sentence presented had a maximum of 22 words. Each word in the sentence was visually presented at 500 ms per word. The 500 ms/word pace was chosen out of a variety of timings previously considered because it was the one that optimized ease of reading, speed, and accuracy in the comprehension of the sentence.

For 180 (75%) of the sentences, a query (yes/no question) about the sentence just read was presented for 4000 ms. The ISIs within and between (sentence+query) items were each 500 ms for a total of 16 s per item. Accordingly, the total time per run was 6 min 24 s (16 s × 24 sentences).

### 2.3. Procedure

The pre-scanning practice session was designed to familiarize the participants not only with the general procedure in the scanner but also with the length of the experimental sentences. In this practice session each participant was exposed to long embedded sentences similar to the ones they would be encountering in the study and at the same reading pace: one word at a time, paced at 500 ms per word, presented at the center of the screen and followed by a comprehension question.

Participants were instructed to read the sentences silently in the most natural way possible. To facilitate this, sentences were presented with punctuation marks (commas) supporting a native prosodic contour. Responses to the queried sentences were recorded with a yes/no button box. The total duration of the functional component of the study was about an hour, and the total duration of the testing session was 90 min.

### 2.4. Participants

Fifteen native speakers of English (8 female and 7 male) between the ages of 18 and 22 participated in this study. All except for one subject were right handed with normal or corrected-to-normal vision. By their own report, none had suffered a concussion nor were they under treatment for a neurological or psychological condition. All participants gave written informed consent in accordance with the guidelines set by the Yale University Human Subjects Committee and were compensated for their participation.

### 2.5. Data acquisition

Head positioning in the magnet was standardized using the canthomeatal landmarks. In the scanner, cushions inside the head coil were used to reduce head movement and headphones were used to dampen the scanner noise and to communicate with participants. Conventional T1-weighted spin-echo sagittal anatomical images were acquired for slice localization using a 1.5T whole body imaging system with a quadrature head coil (Siemens, Erlangen, Germany). After a 3-plane localizer and a multiple-slice sagittal localizer, 28 T-1 weighted axial slices (*TR* = 485 ms; *TE* = 11 ms; bandwidth = 130 Hz/pixel; *FA* = 90°; slice thickness = 5 mm; FOV = 200 × 200 mm; matrix = 256 × 256) were obtained using flash spin-echo imaging parallel to the anterior and posterior commissure (AC–PC). Ten functional data series were then acquired with a single-shot gradient-echo echo planar imaging (EPI) sequence (*TR* = 2000 ms; *TE* = 30 ms; bandwidth = 1735 Hz/pixel; *FA* = 80°; slice thickness = 5 mm; FOV = 220 × 220 mm; matrix = 64 × 64; with 196 measurements) with same slice localizations as the T-1 anatomical. Stimuli were projected onto a semi-transparent screen at the head of the bore, viewed by the subject via a mirror mounted on the head coil. At the end of the functional imaging, a high resolution 3D Magnetization Prepared Rapid Gradient Echo (MPRAGE) sequence (*TR* = 24 ms; *TE* = 4.66 ms; bandwidth = 130 Hz/pixel; *FA* = 45°; slice thickness = 1.3 mm; FOV = 340 × 340 mm; matrix = 256 × 256) was used to acquire sagittal images for multi-subject registration.

### 2.6. Data analysis

All data were converted from Digital Imaging and Communication in Medicine (DICOM) format to analyze format using XMedCon (Nolfe et al., 2003). During the conversion process, the first three images at the beginning of each of the eight functional series were discarded to enable the signal to achieve steady-state equilibrium between radio frequency pulsing and relaxation leaving 193 images per slice per trial for analysis. Functional images were realigned (motion-corrected) with the Statistical Parametric Mapping 5 algorithm (www.fil.ion.ucl.ac.uk/spm/software/spm5) for three translational directions (x, y, or z) and three possible rotations (pitch, yaw or roll). Trials with linear motion that had a displacement in excess of 1.5 mm or rotation in excess of 2 degrees were rejected.

Individual subject data were analyzed using a General Linear Model (GLM) on each voxel in the entire brain volume with regressors specific for each task. For each of the four sentence types (A, B, C, D) there were four regressors (shown in Table 2): **Event 0** = onset of the first word up to the offset of “that/about,” **Event 1, GAP-search** = onset of subject of relative/complement clause up to offset of word before lowest embedded verb; **Event 2, GAP-completion** = onset of lowest embedded verb up to end of the sentence, **Question** = onset of comprehension question up to the end of the question. We account for the hemodynamic delay within the General Linear Model used which includes the waver hemodynamic response function (hrf) from the AFNI software.

The resulting beta images for each task were spatially smoothed with a 6 mm Gaussian kernel to account for variations in the location of activation across subjects. The output maps were normalized beta-maps, which were in the acquired space (3.438 × 3.438 × 5 mm).

To take these data into a common reference space, three registrations were calculated within the Yale BioImage Suite software package (www.bioimagesuite.org, Papademetris et al., 2006). The first registration performs a linear registration between the individual subject raw functional image and that subject's 2D anatomical image. The 2D anatomical image is then linearly registered to the individual's 3D anatomical image. The 3D differs from the 2D in that it has a 1 × 1 × 1 mm resolution whereas the 2D z-dimension is set by slice-thickness and its x-y dimensions are set by voxel size. Finally, a non-linear registration is computed between the individual 3D anatomical image and a reference 3D image. The reference brain used was the Colin27 Brain (Holmes et al., 1998) which is in Montreal Neurological Institute (MNI) space (Evans et al., 1992) and is commonly applied in SPM and other software packages. All three registrations were applied sequentially to the individual normalized beta-maps to bring all data into the common reference space.

Data were corrected for multiple comparisons by spatial extent of contiguous suprathresholded individual voxels at an experiment-wise *p* < 0.05. In a Monte Carlo simulation within the AFNI software package and using a smoothing kernel of 6 mm and a connection radius of 6.97 mm on 3.44 × 3.44 × 5 mm voxels, it was determined that an activation volume of 197 original voxels (5319 microliters) satisfied the *p* < 0.05 threshold. Clusters were created for each of the four subtractions. Each cluster was identified with a region label, and then associated with additional numeral labels corresponding to Brodmann areas. Regional labels were assigned using the Yale Brodmann Area Atlas which is defined on the Colin27 Brain at 1 mm resolution.

### 2.7. Predictions

Table 3 presents the planned single subtractions isolating the two mechanisms in question and corresponding to the two (intrasentential) events: Event 1 and Event 2. Event 1-related subtractions target *GAP-search* and *direct vs. indirect GAP-search*: the correlates of a lexically driven search for the GAP in two contexts, direct vs. indirect, above and beyond phrase-structure building considerations. Event 2-related subtractions target *GAP-completion*: the satisfaction of the syntactic and lexico-semantic requirements of the RELPRO as comprehension unfolds. In addition, a series of *double subtractions* and three conjunction analyses were also performed to show whether or not any of the potential effects observed could be viewed as tapping a common cognitive process and if so which one. The specific double subtractions and conjunction analyses are presented further below in connection to the corresponding general predictions^8^.

If *GAP-search*-which takes place during Event 1- and *GAP-completion*-which takes place during Event 2- place compositionally distinct linguistic demands with presumably different memory load implications, then they are likely to have distinct cortical recruitment commitments. The existence of distinct cortical recruitment is in turn hypothesized to be the root of the lesion-based/neuroimaging incongruence regarding LDD implementation. This distinction in recruitment should be observed between the two events across the relevant conditions (e.g., **Conditions A and B** vs. **Condition D** during Event 1 and **Conditions A and D** vs. **Condition D** during Event 2). Specifically:

#### 2.7.1. Prediction for *GAP-search*: *GAP-search*_*direct*_ and *GAP-search*_*indirect*_

If the LIF cortex supports *GAP-search*, regardless of whether it locally leads to a GAP position or not, both the *GAP-search*_*direct*_ and *GAP-search*_*indirect*_ conditions (**Condition A**, Event 1 and **Condition B**, Event 1, respectively) should elicit the same pattern when a no-GAP condition is subtracted (**Condition D**, Event 1).

If, by contrast, the brain distinguishes between the situation where the memory system is actively *participating* in the *GAP-search* process, rather than simply *supporting* the phrase structure composition that happens to involve this process, we should observe a divergence in activation. In this case we expect that at least *GAP-search*_*direct*_- the condition that has been previously reported to be vulnerable in Broca's aphasia, is correlated with LIF cortex activation.

Three double subtraction analyses (1) *GAP-search* A_1_>D_1_ vs. *GAP-completion* A_2_>D_2_, (2) *GAP-search* B_1_>D_1_ vs. *GAP-completion* B_2_>D_2_ and (3) *GAP-search*_*direct*_ A_1_>D_1_ vs. *GAP-search*_*indirect*_ B_1_>D_1_ and one conjunction analysis *GAP-search*_*direct*_ A_1_>D_1_ and *GAP-search*_*indirect*_ B_1_>D_1_ are relevant for this prediction. The first two double subtractions test LIF cortex sensitivity to *GAP-search* once activation associated with *GAP-completion* has been eliminated. The third double subtraction and the conjunction analysis allows us to see the extent to which *GAP-search*_*direct*_ and *GAP-search*_*indirect*_ have common activation.

#### 2.7.2. Prediction for *GAP-completion*

Our analysis confers *GAP-completion* a subordinate role in LDD composition as it is a strictly local process connecting *GAP-search* to the ongoing composition of the sentence. In terms of cortical localization, we have seen that the previous neuroimaging record does not isolate it. By contrast, the focal-lesion record gives us an important clue as to *GAP-completion*'s potential cortical distribution: For Broca's patients, the reactivation of the GAP, presumably involving *GAP-completion*, is not simply absent, it is *abnormal*. The GAP-filling effect is absent right after the licensing verb, but visible around 500 ms later (e.g., Burkhardt et al., 2003; Love et al., 2008). On the basis of our analysis, we interpret this comprehension pattern as the manifestation of a dissociation between *GAP-search* and *GAP-completion* such that the latter is evidently impacted by, but is not crucially dependent on, the workings of the LIF cortex.

Completing this picture, the lesion-based evidence also tells us that Wernicke's patients are able to implement gap-filling in a timely manner. Yet, *in offline tasks* such as sentence-to-picture matching, these very patients show impaired comprehension not only of object relative clauses, but also of subject relative clauses and non-embedded agentive matrix clauses, a behavior that has traditionally been rooted to a lexically-based deficit, and that accordingly confers Wernicke's area a generalized *compositional* role with direct semantic implications (e.g., Caramazza and Zurif, 1976; Shapiro and Levine, 1990; Piñango and Zurif, 2001, 2015).

Combining these pieces we reason that if there is a connection between LDD composition and the LPST cortex at all, it should be neither in connection to *GAP-search* nor to *GAP-completion* specifically, but in connection to a more general compositional process, involving the coupling of morphosyntactic and semantic composition, of which *GAP-completion* is but one manifestation. So, the localization prediction for *GAP-completion* is exploratory: *GAP-completion*—targeted in three Event 2-related comparisons (1) **Condition B** vs. **Condition C**, (2) **Condition B** vs. **Condition D**, and (3) **Condition A** vs. **Condition D**—should not activate the LIF nor the LPST cortices. But it should show an activation pattern that is instead neuroanatomically *connectable* to both LIF and LPST cortex associated with Broca's and Wernicke's aphasia respectively.

In terms of the double subtraction and conjunction analyses associated with this prediction, our objective is to determine whether or not, despite arising from different contrasts, the three activation patterns predicted to reveal *GAP-completion* indeed manifest the same preferential recruitment. Specifically, we compare A_2_>D_2_ and B_2_>D_2_ to each other and crucially to B_2_>C_2_, and look at how they differ (subtractions) and what cortical recruitment they have in common (conjunction).

In the strongest form of the prediction, if A_2_>D_2_ and B_2_>D_2_ are targeting the same process, subtracting them from each other and from B_2_>C_2_ should result in no difference. By the same token if the three subtractions are revealing the same cognitive process, the conjunction analysis with all three subtractions A_2_>D_2_ and B_2_>D_2_ and B_2_>C_2_ should show a high degree in overlap, one that is coherent with the single subtraction results.

## 3. Results

### 3.1. Behavioral task

Results from the post-sentential questions show an average accuracy rate of 87.6%, which was distributed across conditions as follows: Condition A: 90.57% (29.24), Condition B: 87.47% (33.1), Condition D: 84.82% (35.9). A mixed-model analysis revealed a marginally significant effect of condition [Chi-square = 7.59, (*df* = 2) *p* = 0.083]. Pairwise comparisons revealed a significant difference in A vs. D (*p* = 0.001) and a marginally significant difference in A vs. B (*p* = 0.08). There was no difference between B vs. D (*p* = 0.1) (all results corrected for multiple comparisons).

Given that all conditions show an accuracy rate higher than 80%, we interpret the A vs. D difference as the result of lapses in attention due to the relatively undemanding nature of the D condition which allowed the subjects to lose concentration and in turn miss some of the comprehension questions.

### 3.2. Isolating *GAP-search*: *GAP-search*_*direct*_ and *GAP-search*_*indirect*_

**Figure 1:**
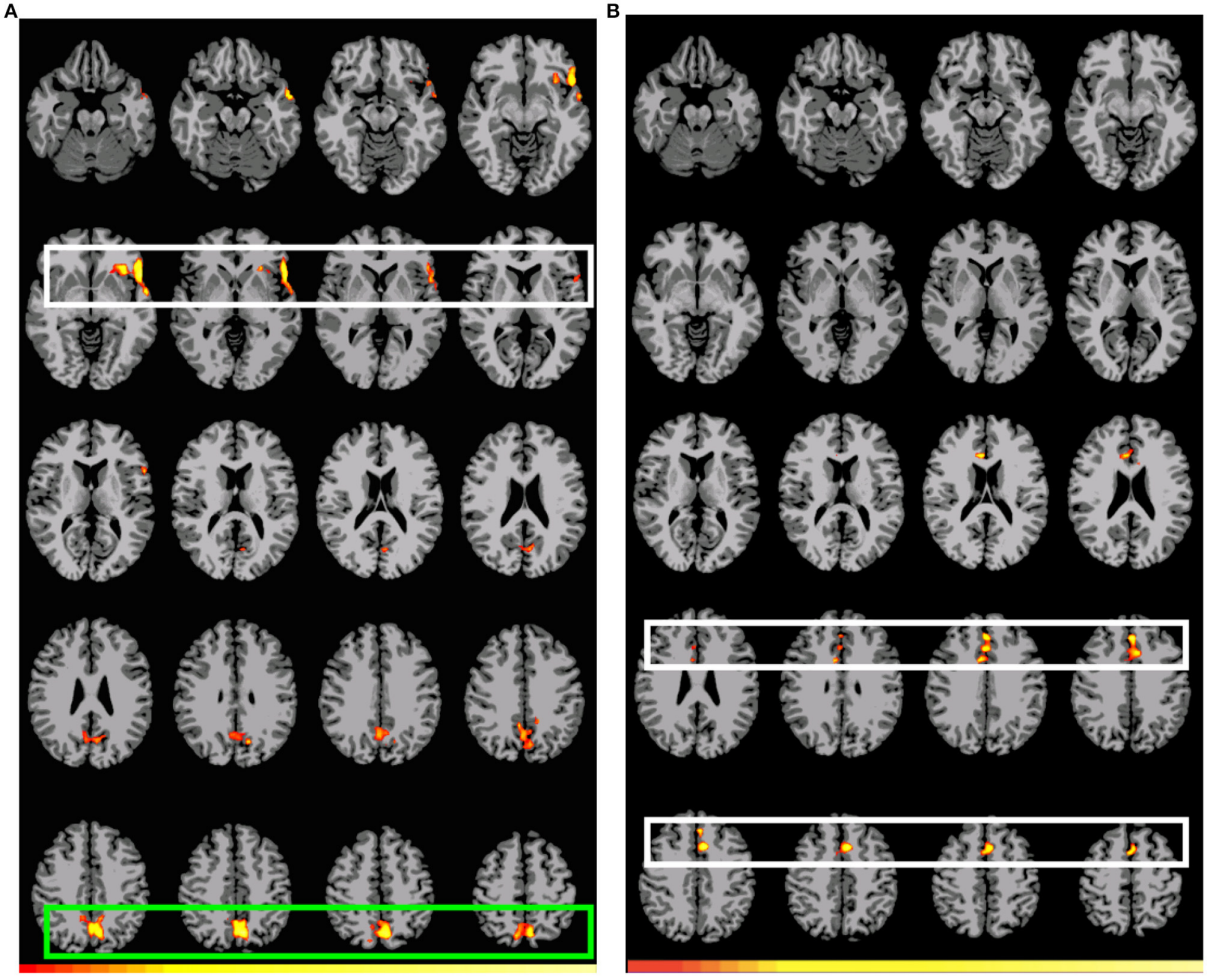
**Preferential activation for both *GAP-search* (Event 1) subtractions**. Images are shown corrected at *p* < 0.05 in radiological format (LH is on the right). **(A)** *(Direct) GAP-search* (Event 1) subtraction: A_1_>D_1_: “the journalist claimed that the government report”>“the journalist's claim that the government report.” White: BAs 45, 44, and 47. Green: Posterior cingulate and sensory association cortex. Low thresholds (*p* < 0.05; *t* = 2.14) are indicated by red, while high thresholds (*p* < 0.000007; *t* = 6.94) are indicated by yellow. **(B)** *(Indirect) GAP-search* (Event 1) subtraction: B_1_>D_1_: “the journalist's claim about the government report”>“the journalist's claim that the government report.” White: SMA activation. Only positive activation reported. Low thresholds (*p* < 0.05; *t* = 2.14) are indicated by red, while high thresholds (*p* < 0.00002; *t* = 6.42) are indicated by yellow.

Figure 1A shows the pattern of activation, presented in radiological format, for the A_1_>D_1_ subtraction (Conditions A and D, Event 1). Two main regions of preferential activation are observed: the first one involves left BAs 45, 44, 47, 22 (inferior, medial), 38, and insula. The second one involves posterior cingulate, left primary and association cortex, and BAs 7 and 31 (both bilateral). Figure 1B shows the B_1_>D_1_ contrast. Interestingly, this pattern of activation appears as a non-overlapping recruitment involving one region connecting bilateral BA 6 (medial superior), bilateral BA 8, and bilateral BA 32 and right BA 24. Table 4 below shows the significant differential volume by region for each of these comparisons.

**Table 4:** **Significant differential volumes by region for *GAP-search* subtractions**.

***(Direct) GAP-search (Event 1) subtraction, A***_1_>***D***_1_				
**Region**	**Volume (mm^3^)**	**Mean** ***T*****-value**	**Max** ***T*****-value**	**Max MNI Coords. (x, y, z)**
Region 1: left BA 45, left BA 44, left BA 47, left BA 22 (inferior, medial), left BA 38, insula	5571	2.94	5.83	−54, 18, −3
Region 2: posterior cingulate, left visual cortex (primary and association), bilateral BA 7 and 31	9516	2.82	6.94	−9, −66, 45
***(Indirect) GAP-search (Event 1) subtraction, B*****_1_>***D***_1_**				
**Region**	**Volume (mm^3^)**	**Mean** ***T*****-value**	**Max** ***T*****-value**	**Max MNI Coords. (x, y, z)**
Right BA 24, bilateral BA 6 (medial superior), bilateral BA 8, bilateral BA 32	6262	2.80	5.56	−6, 15, 45

**Figure 2:**
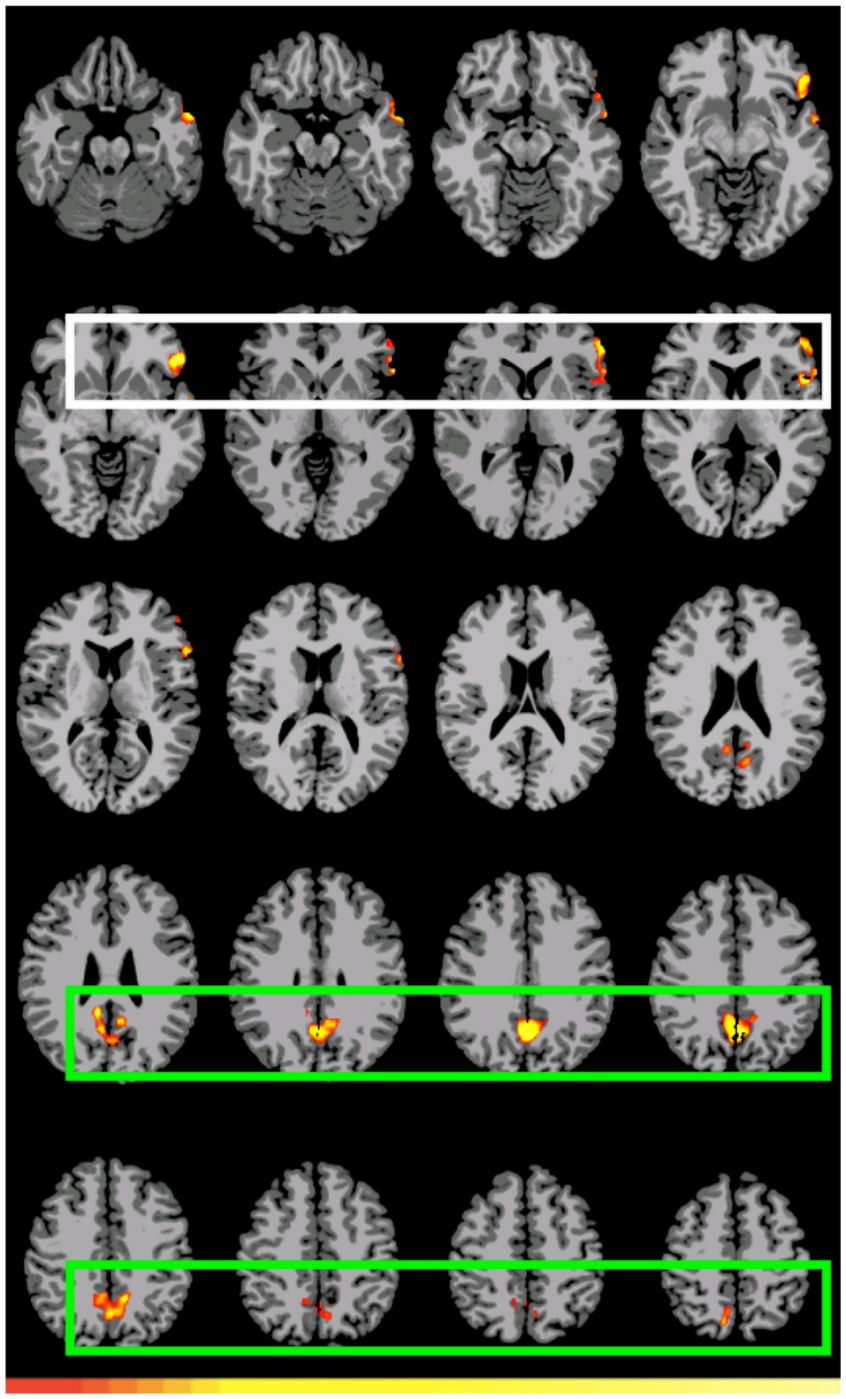
**Preferential activation for *(direct) GAP-search* (Event 1) double subtraction: (A_1_>D_1_) vs. (A_2_>D_2_)**. White: BAs 44, 45, 46, and 47. Green: Posterior cingulate and sensory association cortex. Images are shown corrected at *p* < 0.05 in radiological format (LH is on the right). Only positive activation reported. Low thresholds (*p* < 0.05; *t* = 2.14) are indicated by red, while high thresholds (*p* < 0.000005; *t* = 7.20) are indicated by yellow.

The first double subtraction (A_1_>D_1_ vs. A_2_>D_2_) (Figure 2, Table 5 below) shows a pattern almost identical to the one yielded by the original single subtraction: left BAs 47, 46, 45, 44, and 38, medial BA 7, BAs 17, 18, and 19, and the cerebellum. The second and third double subtractions (B_1_>D_1_ vs. B_2_>D_2_) and (A_1_>D_1_ vs. B_1_>D_1_) by contrast yielded no significant activation.

**Table 5:** **Significant differential volumes by region for *GAP-search* (Event 1) double subtraction (A_1_>D_1_) vs. (A_2_>D_2_)**.

**Region**	**Volume (mm^3^)**	**Mean *T*-value**	**Max *T*-value**	**Max MNI Coords. (x, y, z)**
Region 1 left BA 47, left BA 46, left BA 45, left BA 44, left BA 38 (temporal pole)	9328	2.70	6.03	−51, 30, −6
Region 2: posterior cingulate, sensory (association)	7522	2.94	5.84	0, −63, 36
Region 3: cerebellum	7526	2.71	6.15	24, −69, −33

Finally, the conjunction analysis counterpart comparing direct vs. indirect search showed an empty intersect. This analysis which, crucially, is based only on the corrected maps, tells us that for this comparison the stronger more reliable activation is in terms of the *differences* in preferential activation between the two contrasts. This supports the possibility that for Event 1, any privileged association is not between LIF cortex and *GAP-search* but between LIF cortex and *GAP-search*_*direct*_.

### 3.3. Isolating *GAP-completion*

**Figure 3:**
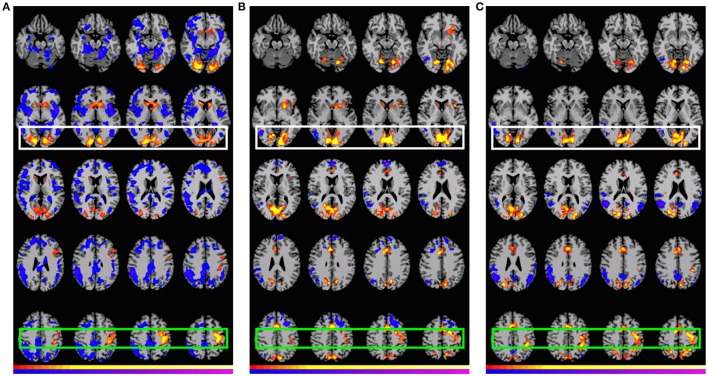
**Preferential (positive and negative) activation for *GAP-completion* (Event 2) subtractions**. White: Visual cortex (association and primary), Green: SMA and parietal activation. Images are shown corrected at *p* < 0.05 in radiological format (LH is on the right). For **(A)**, low thresholds (*p* < 0.05; *t* = 2.14) are indicated by red and blue, while high thresholds (*p* < 0.000000005; *t* = 12.6) are indicated by yellow and purple. For **(B)**, low thresholds (*p* < 0.05; *t* = 2.14) are indicated by red and blue, while high thresholds (*p* < 0.0000006; *t* = 8.6) are indicated by yellow and purple. For **(C)**, low thresholds (*p* < 0.05; *t* = 2.14) are indicated by red (positive activation) and blue (negative activation), while high thresholds (*p* < 0.0000003; *t* = 9.1) are indicated by yellow (positive activation) and purple (negative activation). **(A)** B_2_>C_2_: “had bothered_*gap*_ is calling a press conference.”>“had bothered the people is calling a press conference.” **(B)** A_2_>D_2_: “had bothered_*gap*_ is calling a press conference.” > “had bothered the people and is calling a press conference.” **(C)** B_2_>D_2_: “had bothered_*gap*_ is calling a press conference.” > “had bothered the people and is calling a press conference.”

Table 6 (Figure 3) shows the pattern of activation for all three contrasts involving *GAP-completion*: **Condition B** > **Condition C** (Event 2), **Condition A** > **Condition D** (Event 2), and **Condition B** > **Condition D** (Event 2), respectively. We interpret them together because the pattern of activation they each give rise to is by our hypothesis reflecting the same *GAP-completion* process. We present them separately because each emerges from different surface-level subtractions: a legitimately filled gap vs. an illegitimately filled gap (B_2_>C_2_) and a filled gap vs. non-gap (A_2_>D_2_ and B_2_>D_2_).^9^ Moreover, those segments come from different (non-local) sentential contexts (A and B, respectively). We reason that if *GAP-completion* is an isolable process, it should yield a similar activation pattern regardless of non-local context. This is especially the case for A_2_>D_2_ and B_2_>D_2_ which share the same subtrahend.^10^

**Table 6:** **Significant differential volumes by region for all *GAP-completion* (Event 2) contrasts**.

**Contrast**	**Region**	**Vol. (mm^3^)**	**Mean *T*-value**	**Max *T*-value**	**Max MNI (x, y, z)**
B_2_>C_2_	Region 1: BA 6 (left, medial, bleeding into left BA 44), left primary motor/sensory	17,585	2.80	5.56	−39, −18, 57
	Region 2: visual cortex (primary/association)	28,691	2.80	5.56	15, −87, −6
	Region 3: caudate and putamen	7820	2.80	5.56	18, 21, 6
A_2_>D_2_	Region 1: BA 6 (bilateral, mostly medial/superior, bleeding into BA 8), anterior and dorsal cingulate, left primary motor/sensory	19,052	2.98	7.61	−24, −9, 57
	Region 2: BA 7, visual cortex (primary/association)	45,564	3.13	8.56	−18, −69, −9
	Region 3: caudate, left putamen, left BA 47, left insula	5829	2.63	4.51	−30, 21, 0
B_2_>D_2_	Connected region: BA 6 (bilateral, medial, superior), BA 8, anterior and dorsal anterior cingulate (right), left primary motor/sensory, BA 7 (bleeding into BA 39), left BA 40, visual cortex (primary/association)	72,530	2.97	9.07	−12, −87, −3

Figure 3 shows that there is indeed a very similar pattern of activation across the three subtractions. For all three contrasts, there are two main foci of preferential recruitment: BA 6 (left and bilateral) and visual cortex (primary/association). This said, type of subtraction also mattered: for the A_2_>D_2_ and B_2_>D_2_ contrasts, common preferential areas were revealed which did not emerge in the B_2_>C_2_ subtraction: anterior cingulate, BA 7 (precuneus), and BA 32. In addition, B_2_>D_2_ revealed activation of left BA 40. Finally, none of the contrasts showed overlap with BA 44 or BA 45–regions that were observed in the *GAP-search* condition. This finding was further confirmed in the double subtraction and conjunction analyses (see below). All results (from both Events 1 and 2) are summarized in Table 7 below.

**Table 7:** **Summary of cortical recruitment by Events: Single subtractions**.

**Subtraction**	**Event 1: GAP-search**	**Event 2: GAP-completion**
B > C	–	BA 6 (lateral), BA 44 (edge), visual cortex (BAs 17, 18, 19), caudate and putamen. Activation is also observed in primary motor and primary sensory cortex.
A > D	Region 1: left BA 45, left BA 44, left BA 47, insula, left BA 22 (inferior, medial), BA 38 (temporal pole) Region 2: posterior cingulate, left primary visual cortex, left visual association cortex, bilateral BA 7, BA 31	Region 1: BA 6 (bilateral, mostly medial/superior, bleeding into BA 8), anterior and dorsal cingulate, left primary motor/sensory Region 2: caudate/putamen (left), BA 47 (left), insula (left) Region 3: BA 7 (bilateral), primary visual/association cortex (bilateral medial)
B > D	BA 6 (medial superior), BA 8, BA 24 (right), and BA 32.	BA 6 (bilateral, medial, superior), BA 8, anterior and dorsal anterior cingulate (right), left primary motor/sensory, BA 7 (bleeding into BA 39), left BA 40, visual cortex (primary/association)

**Figure 4:**
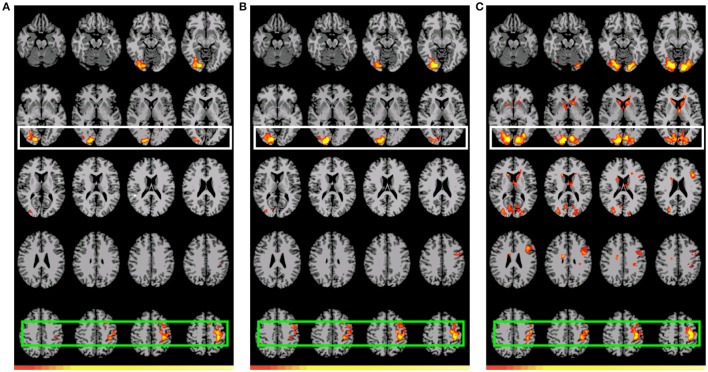
**Preferential positive activation for *GAP-completion* (Event 2) double subtractions**. White: Visual cortex (association and primary), Green: SMA and parietal activation. Images are shown corrected at *p* < 0.05 in radiological format (LH is on the right). For **(A)**, low thresholds (*p* < 0.05; *t* = 2.14) are indicated by red, while high thresholds (*p* < 0.00000001; *t* = 11.8) are indicated by yellow. For **(B)**, low thresholds (*p* < 0.05; *t* = 2.14) are indicated by red, while high thresholds (*p* < 0.000000009; *t* = 12.1) are indicated by yellow. For **(C)**, low thresholds (*p* < 0.05; *t* = 2.14) are indicated by red, while high thresholds (*p* < 0.000000009; *t* = 12.1) are indicated by yellow. **(A)** (B_2_>C_2_) vs. (A_2_>D_2_): Residual preferential activation for *GAP-completion*. **(B)** (B_2_>C_2_) vs. (B_2_>D_2_): Residual referential activation for *GAP-completion*. **(C)** (B_2_>C_2_) vs. (B_1_>C_1_): Preferential activation for *GAP-completion* > preferential activation for *GAP-search*.

**Figure 5:**
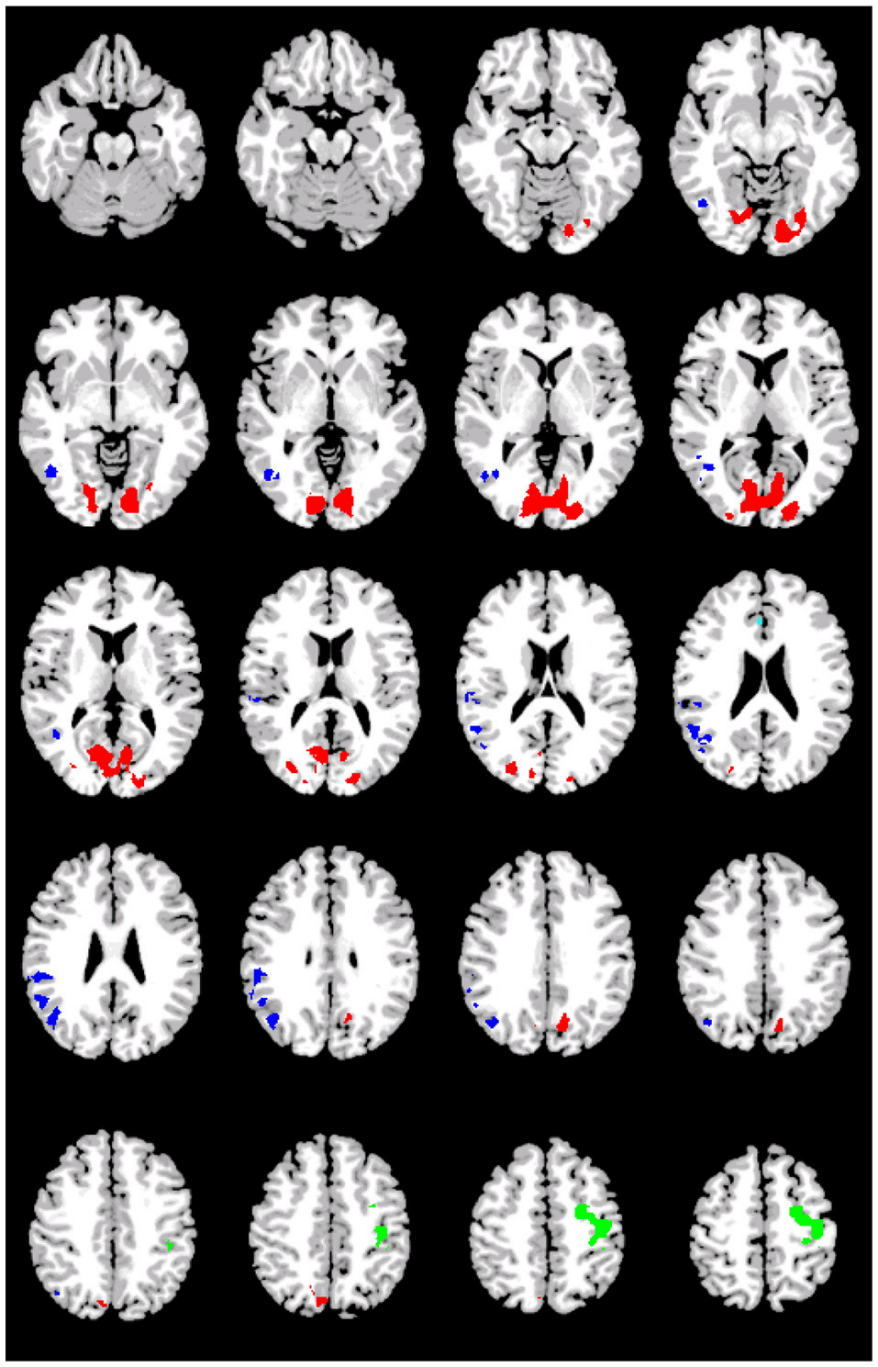
**Preferential activations for the conjunction of *GAP-completion* subtractions**. Images are shown corrected at *p* < 0.05 in radiological format (LH is on the right). Green: BA6, primary motor and primary sensory connected with anterior cinculate (Acqua); Red: BA7, BA19, primary and association visual; Blue: right visual (association), right angular/supramarginal gyri, and fusiform.

The two *GAP-completion*-related double subtractions yielded interesting results. We predicted that if all Event 2 subtractions are targeting the same process, subtracting one from the other should result in no difference. And indeed that is what we found for A_2_>D_2_ vs. B_2_>D_2_. When these two conditions were compared to B_2_>C_2_ a difference was observed not in terms of localization but in terms of *volume* of activation. As Table 8 (see also Figure 4) shows, the activation pattern observed for these single and corresponding double subtractions is almost identical. What we observe in the double-subtraction is a change in the volume for the SMA and which goes from 17,585 in the single B_2_>C_2_ subtraction down to 11,479 when subtracted by A_2_>D_2_ and to 13624 when subtracted by B_2_>D_2_. We compare these double subtractions to one where the *GAP-search* counterpart is subtracted: B_2_>C_2_ vs. B_1_>C_1_. We reason that if the previous two double subtractions are reflecting *GAP-completion* their results should converge with this one which isolates *GAP-completion* from *GAP-search*. And that is what we find. These results are summarized in Table 9. Finally, the conjunction analysis confirms these findings by showing again not only the primary and association visual cortex and connected posterior cortex, but crucially, BA 6 as a main area of overlap. These results are summarized in Table 10 and shown in Figure 5.

**Table 8:** **Significant differential volumes by region for all *GAP-completion* (Event 2) double subtractions**.

**Contrast**	**Region**	**Vol. (mm^3^)**	**Mean *T*-value**	**Max *T*-value**	**Max MNI (x, y, z)**
(B_2_>C_2_) vs. (B_2_>D_2_)	Region 1: left BA 6, primary motor/sensory	13264	3.11	7.18	−33, −18, 60
	Region 2: right visual cortex (primary/association)	10579	3.67	12.07	15, −87, −6
(B_2_>C_2_) vs. (B_1_>C_1_)	Region 1 left BA 6, primary motor/sensory, BA 8	18634	3.19	10.06	−39, −27, 57
	Region 2: visual cortex (primary/association)	35814	3.42	12.05	15, −87, −6
	Region 3: caudate, putamen, thalamus	11848	2.68	5.12	18, 21, 12
(B_2_>C_2_) vs. (A_2_>D_2_)	Region 1: left BA 6, primary motor/sensory	11479	2.97	6.29	−51, −18, 51
	Region 2: right visual cortex (primary/association)	9155	3.35	8.91	18, −84, −12

**Table 9:** **Summary of cortical recruitment for all *GAP-completion* (Event 2) double subtractions**.

**Subtraction**	**Event 2: *GAP-completion***
(B_2_>C_2_) vs. (A_2_>D_2_)	Region 1: left BA 6, primary motor/sensory cortex. Region 2: right primary/association visual cortex.
(B_2_>C_2_) vs. (B_2_>D_2_)	Region 1: left BA 6, primary motor/sensory cortex. Region 2: right primary/association visual cortex.
(B_2_>C_2_) vs. (B_1_>C_1_)	Region 1: left BA 6, primary motor/sensory cortex, BA 8. Region 2: primary/association visual cortex. Region 3: caudate, putamen, thalamus

**Table 10:** **Significant differential volumes by region for conjunction of *GAP-completion* (Event 2) subtractions, (A_2_>D_2_) + (B_2_>C_2_) + (B_2_>D_2_)**.

**Region**	**Volume (mm^3^)**	**Mean *T*-value**	**Max *T*-value**	**Max MNI Coords. (x, y, z)**
Region 1 BA 6, motor (primary, supplementary), sensory (primary), anterior cingulate	5399	X	X	−2, −82, 5
Region 2: right visual (association), right angular/supramarginal gyri, fusiform	4422	X	X	49, −55, 19
Region 3: BA7, BA19, visual (primary, association), sensory (association)	15,869	X	X	−33, −16, 54

### 3.4. Activation beyond *GAP-search* and *GAP-completion*: discourse-composition and GAP violation

In the Event 2 contrast, an additional pattern of activation is observed which results from the inverse subtraction C_2_>B_2_ and which is associated with a GAP violation. (The violation is caused by an expected GAP that already appears filled.) This contrast was not part of the main question the study seeks to address, but in light of the other results, it reveals a very interesting pattern which we believe is connectable to our main question. The C_2_>B_2_ segment, which reflects the violation proper, recruits no LIF, LPST, or parietal cortices. Instead, it recruits the *right hemisphere* BAs 45 and 46 and bilateral prefrontal cortex (BAs 9 and 10).

This pattern is interesting because it reflects cortical recruitment beyond the traditional language areas, suggesting that its impact is outside language composition strictly speaking. Indeed in connection to this observation a reviewer points out, correctly in our view, that this pattern of activation lines up with the so-called default mode network (DMN); a network traditionally associated with resting states or situations where subjects are left to carry out “undirected” thinking. Consequently, the reviewer suggests, these could be an indication that the parser most likely has simply halted the comprehension process.

We agree with the reviewer that to the extent that we do not fully know the impact of ungrammaticality in the process of comprehension, the possibility remains that faced with ungrammaticality, the comprehension system stops tracking linguistic composition altogether, thus allowing the mind to direct thought away from the utterance in question. This said, we would like to propose an alternative interpretation which is connectable with our present aims: that the pattern of preferential activation observed, partially overlapping with the default mode network, directly reflects the specific *discourse-based* nature of the violation in Condition C; a possibility that complements the recruitment pattern involved in gap-search/completion. On our analysis, the violation in Condition C is caused by the inability of the parser to integrate the composed meanings of the embedded and matrix clauses. These clauses are each independently syntactically and semantically well-formed yet cannot be linked with each other. The ill-formedness is caused by the requirement that *GAP-completion* apply at a point in the sentence where it is not allowed to. *GAP-completion* is the process where the referent associated with the antecedent finds an interpretation as a participant in the semantic representation associated with the embedded clause, thus linking the proposition denoted by the embedded clause with that of the matrix clause. In the ungrammatical utterance, *The politician who the journalist's claim about the government report had bothered* ***the people*** *is calling a press conference, GAP-completion* cannot take place because the GAP is already occupied by another NP (*the people*). Consequently, not only is the antecedent (*the politician*) left without a (necessary) interpretation within the embedded clause, but a new and unexpected semantic interpretation (involving the participant *the people*) has been introduced, which is locally plausible but cannot be connected with the meaning of the matrix clause. These two locally coherent segments (matrix clause: *the politician is calling a press conference* and embedded clause: *the journalist's claim about the government report had bothered the people*) result in compositionally conflicting linguistic representations, which in turn yield a meaning incoherence for the sentence as a whole (i.e., two mutually exclusive individuals “the politician” and “the people” must be licensed as the experiencer of “bother”). The meaning of the embedded clause (containing the new participant) can no longer be incorporated into the meaning of the matrix clause (containing the antecedent). This incoherence cannot be resolved not because there is no one plausible interpretation to be obtained, but because there is one too many plausible interpretations.

We propose that the comprehension system is sensitive to this situation and it is the conflict that it represents what underlies the activation pattern observed for C>B event 2. This would suggest in turn that the thrust of the violation lies on higher level meaning-based structure, even though the violation itself is triggered by a local syntactico-semantic misstep^11^.

If this were the case, it would make the non-linguistic regions in question relevant for language comprehension processes involving contextualization or integration of composed meaning. Early support for this possibility is found in fMRI reports suggesting a correlation of relevant right-hemisphere cortical areas with notions such as “discourse” level composition (e.g., Costello and Warrington, 1989; Devlin et al., 2003) and “aboutness” (Bornkessel-Schlesewsky et al., 2012). Specifically relevant to the DNM is the work on fMRI patterns relating the DMN to social cognition processes, in particular those connecting middle frontal cortex with theory of mind processes (see Mars et al., 2012 for a meta-analysis of this body of work in connection also to DMN processes in non-human primates). As noted, this interpretation is not intended to apply to syntactic violations across the board, but to activation patterns where the violation results in a larger discourse incoherence such as that created by a “doubly-filled” argument position. (For a more general discussion about brain patterns and violations, see Embick et al., 2000; Friederici et al., 2003).

## 4. Discussion

Past neuroimaging work has shown that even though long-distance dependencies seem to recruit the workings of the LIF cortex, they also recruit the workings of the LPST cortex and surrounding areas (e.g., Cooke et al., 2002; Fiebach et al., 2002; Amunts et al., 2004; Fiebach et al., 2005; Grodzinsky and Friederici, 2006; Santi et al., 2015). Moreover, while the lexical role of the LPST cortex has been well documented (see Wise et al., 2001; Hickok and Poeppel, 2004, 2007 for proposals regarding the role of the various subcomponents of the LPST cortex in long-term phonological encoding), no conclusive explanation has been given for why this area should be recruited in the instantiation of these dependencies. At the same time, whereas Wernicke's patients (with damage involving the left posterior temporal cortex, including parts of the angular and supramarginal gyri) show across-the-board impaired sentence comprehension including constructions containing dependencies, they are indistinguishable from matching controls in their ability to exhibit the gap-filling effect, thus indicating that whatever their linguistic impairment, it does not seem to involve *GAP-search* or *GAP-completion per se*.

Indeed, Wernicke's performance has been seen to reflect the capacity to implement the basic syntactic mechanics of the dependency, but showing, offline, an inability to put this knowledge to use, presumably due to an inability to properly access the necessary lexico-semantic information that makes the dependency meaningful (e.g., Caramazza and Zurif, 1976; Shapiro and Levine, 1990; see Piñango and Zurif, 2015 for a summary of the main findings). By contrast, Broca's patients, while unable to properly implement these dependencies (e.g., Zurif et al., 1993, 1994; Burkhardt et al., 2003; Love et al., 2008), show, offline, a selective pattern of impairment whereby canonical (subject) relative clauses result in above-chance performance and non-canonical (object) relative clauses reliably result in poor (chance-level) comprehension, a pattern of performance that appears to be linguistic in nature. So whereas the neuroimaging evidence tells us the brain regions that could be potentially participating in the implementation of the dependencies, the lesion-based evidence tells us of the possibility of an *asymmetry* in their participation.

The analysis of LDDs that we present here provides the basis for a potential reconciliation of these two sets of seemingly conflicting observations by invoking organizing principles that could give rise to such an asymmetry. Specifically, the model captures the main linguistic components of a dependency (phrase structure building, argument structure licensing, and pronoun resolution) as selectional/subcategorization constraints on the relative pronoun that separate the process of searching for the environment of argument licensing within the sentence (*GAP-search*) from the actual argument licensing (*GAP-completion*).

In the remainder of this section we discuss the specific activation patterns observed in connection to the hypothesized functional distinctions.

### 4.1. *GAP-search*: *GAP-search*_*direct*_ vs. *GAP-search*_*indirect*_

The hypothesis that LIF cortex is sensitive to *GAP-search* independently of the internal articulation of the dependency (direct vs. indirect) was not borne out. To the extent that *GAP-search* was reliably associated the LIF cortex it was only in connection to the *direct* condition (single and double subtractions). Within this pattern of activation two connected regions were involved: region 1 included BAs 45, 44, 47, bordering with the left insula and left temporal pole (anterior BAs 22 and 38). A second associated region connecting primary and associate visual cortex and BA7 and BA31 were also preferentially recruited. This second region of activation is interesting for two reasons; (1) it appears in A_1_>D_1_ but not in the B_1_>D_1_ contrast, and this is relevant because it involves the participation of BA7, a cortical region previously connected to CP embedding, precisely the kind of composition present in A_1_ and absent in B_1_, and (2) it continues to appear in connection to *GAP-completion* for both A_2_>D_2_ and B_2_>D_2_ contrasts, thus suggesting that this area is sensitive to general composition such as that involved in *gap-completion*^12^.

The results from the A_1_>D_1_ vs. A_2_>D_2_ double subtraction, support the importance of the LIFG for *GAP-search*_*direct*_, an observation that replicates previous findings both from neuroimaging and lesion-studies. Those results further indicate that this cortical recruitment may at least be partly distinct from the cortical recruitment of *GAP-completion*.

Results also show that when *GAP-search* encounters a linguistic “obstacle”—as in Condition B (Event 1) *GAP-search*_*indirect*_, and revealed in the B > D (Event 1) contrast - a different preferential activation pattern emerges involving BA 6 (medial superior), BA 8, right BA 24, and BA 32. At the same time, results from the double-subtraction A_1_>D_1_ vs. B_1_>D_1_ reveal no preferential activation suggesting that these two conditions are also very similar. So, in light of the ambiguous statistical results, we offer an interpretation constrained by previous neuroimaging and lesion-based observations. We propose here that these two sets of results indicate there may not be a categorical distinction between the cortical regions engaged in *GAP-search*_*direct*_ vs. those engaged in *GAP-search*_*indirect*_, instead the two reflect different patterns of activation within what is ultimately the same cortical network.

We thus interpret the LIF cortex preferential activation associated with *GAP-search*_*direct*_ as resulting from an *interaction* of two factors involved in LDD resolution: (a) the prediction of a GAP, and (b) the possibility that the GAP be found within the syntactic and semantic contexts immediately after the RELPRO, that is, when nothing in the unfolding syntactic and semantic structure prevents the licensing of the GAP. These findings would thus represent independent neurological support for the existence of an active-filler (Clifton and Frazier, 1989; Frazier and Clifton, 1989; Fodor, 1995) that, crucially, is sensitive to the details of the linguistic context of the relative pronoun independently of the length of the dependency (Phillips et al., 2005).

Indeed, we take this pattern to reflect not necessarily a difference in search but a difference in quality of the search: when the parser is forced to use memory resources outside of the implementation of any specific linguistic mechanism -the delay caused by the parser's recognition that the expected GAP is *not* to be found in the current local constituent- those resources are recruited from cortical regions, most relevant BA 6 (SMA), which have been previously identified as participatory for language composition. The combined *GAP-search* pattern of results (direct plus indirect) would thus be reflecting the workings of two functional foci of the same linguistic *network*.

Support for this view is the observation that the LIF cortex and SMA have been traditionally connected, particularly in the focal-lesion literature (e.g., Benson, 1985; Tonkonogy, 1986; Vignolo, 1988; Naeser et al., 1989; Alexander et al., 1990; Goodglass, 1993). This would mean in turn that the LIF cortex is sensitive to the expedient resolution of the dependency, which will only happen when such resolution is allowed by the local linguistic context. If it is not, then the preferential activation shifts (or reduces) to pre-SMA–all, however, within the same pathway.

This interpretation is consistent with Santi and Grodzinsky (2012) regarding the connection between “prediction” and the LIF cortex. Yet, what our results show is that presence of “prediction” is not enough. For the LIF cortex to be fully engaged, it must continuously be tracking for “gap-viability” as composition unfolds^13^.

Further elaborating on this issue, a reviewer suggests a perspective on the B_1_>D_1_ activation pattern that gives it a specific role—namely the suppression or inhibition of the direct *GAP-search* mechanism associated with the LIF cortex. In this view then, the monitoring action would presumably rely on the workings of the pre-SMA and in the situation where the *GAP-search* could not take place, due to the island, it would act on the LIF cortex to suppress or hold search activity. We agree that this possibility, though outside the scope of the present data, is interesting and consistent with all other roles independently attributed to the SMA (e.g., Schwartze et al., 2012). Moreover, it brings the debate not only to a discussion of networks but to the possible distinguishable roles that their individual components may play during real-time cognitive processing.

Indeed, we take the activation of the supplementary motor area (SMA) in the B>D (Event 1) contrast to be an important clue to the cortical recruitment of LDDs. Not only regarding *GAP-search*, but also *GAP-completion* as we will see below. Specifically, pre-SMA and SMA-proper (BA 6) have been independently shown to be involved in sensory-motor processing possibly manifested through a “gradient” in which sensory, nonsequential, suprasecond information is processed rostrally (recruiting pre-SMA cortex) while motoric, sequential, and subsecond information is processed more dorsally (Schwartze et al., 2012). Our present data are not fine-grained enough to reveal a dissociation between pre-SMA and SMA proper. However, the data do show the shared locus of activation to be on medial BA 6, suggesting the targeting of pre-SMA over SMA-proper. Such a locus would be consistent with the processing of non-motoric, non-sequential, suprasecond information such as that involved in the holding of the filler in memory, as it were, until the “GAP-unviable” segment has passed and active search can resume^14^.

### 4.2. *GAP-completion*

Regarding *GAP-completion*, our findings from the simple subtractions show that this mechanism recruits the workings of a contiguous cortical region within the left fronto-parietal lobes (and non-overlapping with those associated with (direct) *GAP-search*) connecting supplementary motor area, precuneus, and portions of the left angular and supramarginal gyri and peristriate (BA 19). This observation is further supported by all relevant double subtraction and conjunction analyses. What emerges then is a coherent language “network,” as all of these areas have been independently connected with related components of language processing. Most critically, they have been associated with lexically-driven composition, such as that involving subcategorization (Shetreet et al., 2009) and lexico-semantic selectional restrictions (e.g., Lai et al., 2014). Indeed, we conjecture that this pattern of preferential activation is part and parcel of the “Dorsal Stream” or “Dorsal Pathway” (Hickok and Poeppel, 2004, 2007; Friederici, 2009, 2012), which connects the frontal and left posterior cortices via the parietal lobe. To the extent that this network is seen to be involved in a mechanism such as *GAP-completion*, a mechanism that brings together syntactic, lexico-semantic, and discourse composition, it tells us that this cortical region is at least partly recruited during unification of interpretation. And this would also be consistent with a version of the Memory, Unification and Control model (e.g., Hagoort, 2005, 2014) whereby the true locus of *semantic* unification includes, most crucially, at least the pre-SMA. It is in this way that the LPST cortex is connected to LDD implementation: as a potential participating region in a larger network that supports real-time lexically-driven language composition which, by definition, also supports *GAP-completion*.

One additional advantage of the connection between *GAP-completion* with the dorsal pathway is that it affords a possible explanation for the long-standing observation regarding Conduction aphasia comprehension first reported in Caramazza and Zurif (1976). Specifically, Caramazza and Zurif (1976) report that patients with Conduction aphasia (a syndrome associated with damage to the arcuate fasciculus) exhibit *chance* performance in the comprehension of semantically reversible (object) relative-clauses. Such a pattern is indistinguishable from that shown for Broca's comprehension but claimed to emerge from different causes. Caramazza and Zurif (1976) further note that, like Broca's, the pattern shown by Conduction patients contrasts sharply with that exhibited by Wernicke's patients, who show performance that is not attributable to any one linguistic or processing factor. Here we reason that if *GAP-completion* is dependent on the workings of the dorsal pathway, presumably connected to the arcuate fasciculus, it explains why Conduction patients would be impaired in the interpretation of semantically reversible relative clauses, despite being able to carry out *GAP-search*^15^. In sum, we take the overall pattern accrued for all three Event 2, related double subtraction and conjunction contrasts to reflect components of this dorsal pathway, with BA 6 as a crucial area. This interpretation captures the normal-like performance by Wernicke's in online gap-filling constructions and suggests in turn that the LPST cortex activation from the imaging literature may not have been in connection to *GAP-search* proper.

In light of these findings, we are now able to address the questions posed in the introduction. What is the neurocognitive relation between *GAP-search* and *GAP-completion*? Answer: Their loci appears to be the LIF cortex and the (pre-)SMA, respectively. Do they rely on the workings of overlapping brain regions? Answer: The patterns we report show minimal overlap in recruitment. However, to the extent that at least the lower SMA has been considered to be part of Broca's area, they are expected to functionally overlap. Our conjecture regarding the two areas [viable resolution (LIF cortex) vs. holding in memory (SMA)] is a proposal about how this overlap could take place. Can we associate *GAP-completion* to the LPST cortex, thus addressing the lesion-neuroimaging incongruence? Answer: we can if we understand Wernicke's area not as an isolated “language area” but as a part of a larger connectivity pathway “the dorsal stream” that connects Wernicke's area to the left fronto-parietal cortex including BA40, BA7 and the SMA. In line with the lesion-based literature, we conclude that LDD processing (defined in terms of *GAP-search* and *GAP-completion*) does not directly involve the preferential workings of Wernicke's area, but relies on areas that are functionally related to Wernicke's area.

Finally, if the effects reported reflect *GAP-search*, why are they observed mainly in the context of *object*-relative GAPs? Answer: what we find is that the effects reported reflect only an aspect of *GAP-search*, namely the requirement that the RELPRO be locally interpreted. But this only happens when *GAP-search* is being carried out over viable structure. So, it is as if the function of the LIF cortex is to monitor or keep track of the ability of the structure being composed to provide a GAP slot. In terms of our analysis, that amounts to keeping track of whether the selectional requirements of the RELPRO are being satisfied. As long as the composition signals that the GAP is incoming, the LIF cortex is fully engaged. From this perspective, then, the fact that this is observed mainly in object-GAP constructions is not a consequence of the grammatical feature *per se*, but of the fact that in these constructions, it takes longer for the RELPRO to be resolved as compared with subject-gap constructions, thus increasing the probability that the effect will be observed.

As a separate observation, our results also show that processing of memory-taxing sentential constructions (A and B) appear to systematically recruit the workings of the visual cortex (primary and association) areas (see Santi et al., 2015 and references therein for similar findings). We interpret this pattern separately for two reasons: (1) these areas are not traditionally associated with linguistic processing proper, and (2) this preferential activation was observed both during *direct GAP-search* and *GAP-completion*, suggesting that the areas in question are not showing sensitivity to a specific linguistic process.

In light of this, we connect these findings to independent observations regarding the visual system and linguistic load, particularly in relation to pupillometry measures (see Piquado et al., 2010 for a review and additional experimental evidence in relation to language processing load and the visual system). That observation has been shown not to be restricted to cognitive effort, but to extend even to physical effort (Zénon et al., 2014). Accordingly, we take the visual cortex activation pattern to reflect the increased attention (i.e., effort) that the implementation of the relevant linguistic tasks represents but whose source may not be strictly linguistic (see (Martínez et al., 1999; Posner and Gilbert, 1999; Petersen and Posner, 2012) for observations specifically regarding non-visually related attention load and its impact on the visual cortex). In this respect we note that the visual cortex activation was not observed during *indirect GAP-search* further supporting the possibility that during the building of structure that is non-viable for a GAP, no search is actually taking place. And this would make this segment of comprehension less cognitively taxing.

## 5. Conclusions: the present results in the context of neurocognitive architectures

In this section, we connect our results to larger neurocognitive architecture models. In this respect, we consider three models which address syntactic and/or semantic composition, the sort presumably directly involved in *GAP-search* and *GAP-completion*. The first general observation is that whereas no one model accounts for the findings, each provides an insight into the larger pattern that the findings reflect. This gives us, then, the opportunity to focus on the common ground that each provides. This is what guides our discussion.

We start with Lau et al. (2008), who propose a model of semantic composition that could potentially involve LDD composition. In this model, the LIF cortex is connected to lexical retrieval. Interestingly our processing analysis of LDDs is lexically driven, and the key Event 1 contrast A_1_>D_1_ does vary the presence of the relative pronoun. However, as we have seen, lexical retrieval differences alone do not account for the activation pattern: specifically, the results from B_1_>D_1_, which also differ by the presence of the relative pronoun, do not show preferential LIF cortex activation. So, what is required in this model is a more precise treatment of the connection of lexical-retrieval to *GAP-search* in particular^16^.

The second model we consider is Friederici (2012), which proposes that language composition, understood as the process of building a semantic representation through syntactic structure, recruits the workings of the LIF cortex. To the extent that *GAP-search* has been isolated from syntactic structure building through the subtraction process, the model predicts the LIF cortex will not be involved in this process, a prediction that is not supported by the evidence. For the same reasons, the model does successfully predict the absence of activation of BAs 44 and 45, particularly BA 44, in B_1_>D_1_, which involves *GAP-search* but no hierarchical building. Friederici's (2012) model predicts no direct *GAP-search* in connection to the LIF cortex, because according to this model BAs 44 and 45 in particular are responsible for all syntactic structure building. Our results do not contradict this, but do point to the fact that BAs 44 and 45 must be additionally characterized as having specific compositional sensitivity, beyond generalized structure building. Finally, and as mentioned in the discussion, a most relevant aspect of Friederici's (2012) model (which also incorporates important insights from Hickok and Poeppel, 2004, 2007) involves the dorsal pathway, specifically, Pathway I (also discussed in Friederici, 2009) and which connects the STG and BA 6 through the arcuate fasciculus. It is this pathway, we propose, that is responsible for compositional processes such as those represented by *GAP-completion*.

We reserve the end of this section for discussion of the Memory, Unification and Control (MUC) Model (e.g., Hagoort, 2005, 2014). To our knowledge, this is the only model that explicitly assumes lexically-driven processing and grammatical systems, a feature that our processing analysis of LDDs also assumes. The model also capitalizes on the notion of unification, which provides a processing-friendly approach to composition. Like the Friederici (2012) model, Hagoort's 2005, 2014 model proposes a divide within the LIF cortex separating BAs 45 and 47 from BAs 44 and 45 for semantic and syntactic unification/processing functions, respectively. Whereas our data do not speak to the functional articulation within the LIF cortex, they do reveal that both subregions can at least work in tandem, as in the case of the activation for direct *GAP-search*. This is a reasonable interpretation, given that direct *GAP-search* involves both semantic and syntactic computations. What is not clear at this point is how unification should be understood such that it will include *direct GAP-search* as a mechanism while simultaneously excluding *indirect GAP-search*; both processes that are on the one hand “dynamic” in nature, and on the other highly sensitive to the linguistic context of the GAP. Another pending question is the nature of the connection between the LIF cortex and SMA/lower parietal cortex. Under MUC, these two regions could be involved in the same larger processing network, and the SMA activation observed could be part of the dynamics of the network triggered in turn by the linguistic properties of the sentence. In this interpretation, LDDs allow us to localize not two regions, but a network with two foci reflected in these two mechanisms. Since our data cannot speak directly to this point, this proposal remains to be supported.

To conclude, the results presented here suggest a resolution of the imaging vs. lesion incongruence by showing the privileging of BAs 45, 44, and 47 (over BA 6 and parietal and parieto-temporal cortex, including the LPST cortex) in the process of *direct GAP-search* and by suggesting that the activation of LPST cortex reported in the neuroimaging literature is a manifestation of the workings of a network that supports other linguistic compositional processes associated instead with *GAP-completion*.

The results capture the inherent asymmetry between *GAP-search* and *GAP-completion* and explain why damage to the LIF cortex would dramatically impact the ability of the comprehension system to complete the dependency, even if the cortical regions involved in *GAP-completion* remained intact. By the same token, to the extent that the evidence presented here does *not* involve the left posterior superior temporal cortex at least directly, the results tell us why Wernicke's patients should not have issues in searching for and completing the GAP. Indeed, if our conjecture regarding the functional commitments of the SMA and the left lower parietal region (associated with *GAP-completion*) to compositional unification is correct, Wernicke's patients, who have been shown to have lexical retrieval problems, should not show problems in finding/completing the GAP but in unifying this information with the matrix clause into an interpretable string. Such a situation would lead to across-the board comprehension problems in these patients, a prediction that evidence from offline comprehension of these patients (in contrast to Broca's patients) consistently supports.

## Author contributions

MP is responsible for the conception of the work and participated in each aspect of the project including experimental design, data acquisition and analysis, and drafting of the work. EF is responsible for stimuli generation and norming, subject recruitment, data acquisition, data analysis planning (e.g., timing file generation), interpretation and drafting of the work, and participated in the final approval of the version to be published. CL carried out all the data analysis and participated in the final approval of the version to be published. RC participated in the experimental design, data analysis and interpretation, drafting of the work, and in the final approval of the version to be published.

## Funding

This project was funded by NSF BCS-0643266 awarded to MP and NSF-INSPIRE 1248100 awarded to MP, Ashwini Deo, Mokshay Madiman, and RC.

### Conflict of interest statement

The authors declare that the research was conducted in the absence of any commercial or financial relationships that could be construed as a potential conflict of interest. The reviewer WM and handling Editor declared their shared affiliation, and the handling Editor states that the process nevertheless met the standards of a fair and objective review.

## Footnotes

^1^This linguistic description captures the consensus among a variety of syntactic approaches, e.g., Government Binding/Minimalism, Head-Driven Phrase Structure Grammar, Lexical Functional Grammar, and Simpler Syntax, among others, that LDDs are grounded on two organizational properties of language: (1) the possibility to “package” the semantic and syntactic local conditions of the relative pronoun as lexicalized content in the form of subcategorization and/or selectional restrictions, and (2) the possibility of a GAP, a phonologically empty lexico-syntactic entity whose purpose is to instantiate the lexical requirements of the embedded verb; requirements that are expressed in the form of argument structure and subcategorization specifications. These are fundamental and widely accepted properties of the language system. The description presented here is therefore compatible with any representational analysis of relative pronouns that incorporates these two properties (see Culicover and Jackendoff, 2005, for extensive discussion of the syntax-semantics interactions in LDDs and the assumptions that lead the various approaches in question to favor one specific implementation over another).

^2^The index alignment shown across constituents in the syntactic, semantic, and discourse layers makes explicit the observation by most linguistic frameworks of a robust correlation between syntactic category/position, grammatical relation, and semantic relation, such that if a predicate licenses, say, an agent argument, this argument will bear the subject function, which in English can be associated with NP category and SPEC;IP position (e.g., Chomsky, 1965, 1981; Bresnan, 1982, 2001; Fillmore, 1988; Goldberg, 1995; Van Valin and LaPolla, 1997; Culicover and Jackendoff, 2005).

^3^Our selectional criteria, necessary for our localizational purposes, had the unintended consequence of filtering out reports that have otherwise been valuable for our understanding of LDD processing. Fiebach et al. (2005), for example, connect (non-canonical) *GAP-search* to the LIF cortex, but report activation in other areas as well. For their Long > Short (obj.) contrast, they report in addition to the LIF cortex, right inferior frontal (RIF) cortex, junction of the left precentral sulcus, bilateral STS, MTG (21/22) and the left thalamus. By contrast, for their Long > Short (subj.) contrast, no LIFG is reported. Instead, they report activation in the bilateral inferior portion and left superior portion of the parieto-occipital sulcus (BA 17/30 and BA 7 respectively). So, this report relates the LIF cortex to *GAP-search* but not in a unified manner. Similarly, the results published in Makuuchi et al. (2009) address LDDs but are not directly relatable to our present objectives. Whereas they do report LIF cortex activation in connection to comprehension of double-center embedded clauses vs. single-embedded clauses akin to that reported by Santi and Grodzinsky (2007) and Fiebach et al. (2005), their report is based on a region of interest analysis exclusively, and not on a whole brain analysis. Whereas this approach makes sense given their specific interest in the internal articulation of the LIF cortex and not on localizing LDDs components, it prevents us from concluding whether the association they found targeted specifically the LIF cortex.

^4^We do observe, though, that this association is not unambiguous. An alternative interpretation to these findings could be that the reported LIF cortex effect results instead from the composition of a more complex meaning structure associated with a semantically more informative embedded subject. In this scenario, preferential activation of the LIF cortex emerges not from greater LDD distance, but from the semantic demands of processing an incrementally more elaborate embedded subject in composition with the embedded transitive verb and its complement. Indeed, this kind of effect is connectable to a similar LIF cortex recruitment found by Husband et al. (2011) and Lai et al. (2014), who independently show LIF cortex involvement in connection, this time, to the processing of complement coercion (e.g., *The girl began the book* vs. *The girl wrote the book*), a phenomenon also described as involving “enrichment” of the semantic representation.

^5^In another related paper, Santi et al. (2015) test a distinction similar to the one reported in 2007. In addition to the NP category, they introduce CP as potential intervening category. Their results show that for both conditions together (CP+NP), there is, in addition to LIF cortex activation, RIF cortex activation, again correlated with distance. The novel comparison here is the joint results involving the CP condition which, as the authors point out, suggest that the syntactic category of the intervening material is not relevant to *GAP-search*, a conclusion that contrasts with previous findings regarding Broca's poor performance in CP production, and fMRI results showing CP processing in connection to the LIF cortex (Shetreet et al., 2009). As in the case of Santi and Grodzinsky (2007), we believe that their results warrant consideration of an alternative interpretation: the possibility that the increased cost contributed by the CP distance be due instead to the possible garden-path created by the absence of complementizer in the lower CP. In the sentence “I knew [which porter the neurosurgeon said] CP_2_ [the resident liked GAP] CP_1_” two possible structural paths are possible at CP_2_. Specifically, the CP_2_ verb “said” subcategorizes for both an NP and a CP. When an NP is suggested (due to the absence of the complementizer), the CP possibility is discarded. But this soon proves to be the wrong decision both on semantic grounds (the neurosurgeon said [the resident]_*NP*_) and on syntactic grounds (^*^the neurosurgeon said [the resident liked]_**NP*_). Once the parser gets the lower verb “liked,” it must revise its original decision in favor of the CP option, consequently incurring a cost.

^6^We find this kind of comparison to be right-minded and useful also because it connects with independent work on the neurology of anaphora resolution which notably reports an impairment in pronoun and logophor resolution in Broca's patients (e.g., Grodzinsky et al., 1993; Avrutin, 1999; Pinango, 2003; Piñango and Burkhardt, 2005; Schumacher et al., 2010).

^7^We call this condition “indirect *GAP-search*” and not “island“ for the following reason: the term *island* refers to the perspective of the “moved” constituent *before* it has moved. This perspective states that such constituent cannot “leave” the larger constituent in which it is base-generated. To be sure, indirect *GAP-search* is a direct consequence of “movement”; but movement itself is only a metaphor, it has no processing status (i.e., the processor never carries out the movement; it only deals with its consequence). By contrast, the term indirect *GAP-search* is meant to refer to the perspective of the processor (left-to-right incremental composition). For the processor, what matters regarding any type of island is whether upon encountering a given constituent, it can hypothesize that the GAP is to be found within that constituent. If it can, then that constituent is searched for potential GAP positions, if it cannot, then the processor “waits,” as it were, for that (local) constituent to end in order to continue the search. It is this situation that gives rise to the indirectness we refer to: the GAP is incoming, but not in the (minimal) constituent under construction. The “indirect *GAP-search*” label thus allows us to separate the linguistic intricacies of islands, which go well beyond the condition tested here, from one well-attested processing consequence of them. The label “indirect” therefore speaks to the fact that the subcategorized CP is not provided within the local constituent directly after the relative pronoun. So, from the perspective of the parser an “island” is simply a constituent that is not subcategorized and therefore it is not expected to contain the GAP.

^8^We thank a reviewer for calling our attention to the importance of these two second-order analyses which as will be seen strengthened the quality of the evidence overall.

^9^A reviewer asks us about the meaningfulness of the B>C (event 2) comparison. The results from the B>C (event 2) subtraction reflect the preferential activation triggered by B (event 2) without the material that is represented by C (event 2). We infer that this preferential activation includes any process involved in the interpretation of the GAP that survives the violation which, by our analysis, includes the interpretation of the GAP by means of the dependency formation. Indeed, we take this to be the substance of the residual of the subtraction. This contrasts with the violation condition, where the GAP position has been independently filled thus preventing dependency from being completed. As we discuss here, what is interesting of this subtraction, B>C (event 2), is that despite emerging from a subtraction by a violation, the resulting pattern is comparable to the others in Event 2, particularly in terms of the SMA and parietal activation pattern, which the other two non-violation based contrasts also show.

^10^The activation in blue associated with the violation C segment, C_2_ vs. B_2_, is addressed in the subsection “Other Patterns.”

^11^This interpretation rests on a very specific assumption about the parser. The assumption is that the parser will attempt to build an interpretation even in the face of partial incoherence in the input as is the case in condition C. Yet as a reviewer correctly points out this assumption is not necessarily settled in the literature.

^12^A reviewer asks us about our predictions for event 0. We note that no A/B > D difference was predicted and no difference was found at this segment. There are two reasons for this: (1) this early in the sentence, both A/B and D conditions show composition between the head noun and RELPRO, on the one case, and between the subject and the verb, on the other. Even though the nature of the composition that each carries is presumably different, we have no reason to expect that each will recruit visibly distinct cortical regions as a result. (2) Regarding the relative pronoun, even though it is true that by our definition *GAP-search* is triggered as soon as the RELPRO is retrieved, at this early point no structure has been built over which the search is to be carried out. So, even though *GAP-search* is triggered at event 0, it will not be visible until the embedded clause is beginning to be built. This is precisely what the event 1 contrast is intended to reveal.

^13^These findings also connect directly to the cause of the abnormally delayed gap-filling observed in Broca's real-time comprehension (e.g., Burkhardt et al., 2003, Love et al., 2008). The combined behavior of A>D and B>D suggests that Broca's impairment may not be rooted to a generalized problem in syntactic structure formation (brought about in turn to a slowing in lexical retrieval), as has been proposed, but instead to the inability to engage the filler in an active manner as composition progresses, that is, to keep track of the viability of the syntactico-semantic structure being built.

^14^Interestingly, BA 6 and supplementary motor cortex have both been associated to Broca's aphasia *and* Transcortical Motor Aphasia, indicating a potential connection between the two syndromes, whose functional implications are still not well understood (Naeser et al., 1989; Alexander et al., 1990).

^15^The authors thank Julius Fridiksson (p.c) for reminding us of this long-standing yet unexplained observation.

^16^Relatedly, the more natural association with the workings of LDDs would be composition, since after all, the whole motivation for *GAP-search* is to compose the subject matrix nominal into the meaning of the embedded clause (and vice versa). But in the semantic model presented in Lau et al. (2008), this task is connected instead to the anterior temporal cortex (ATC) and angular gyrus, areas that are connectable instead to the ventral pathway and which, in our data, were only partly connected to *GAP-search* (in the form of the marginal activation of the temporal pole for A_1_>D_1_).
