# Supplementary material for: The Localization of Long-Distance Dependency Components: Integrating the Focal-lesion and Neuroimaging Record
Source: Front Psychol. 2016 Sep 30;7:1434. doi: 10.3389/fpsyg.2016.01434 (PMC5043422; doi:10.3389/fpsyg.2016.01434)
Supplement: Supplementary file 1 [file Image1.pdf]

1

The Localization of Long-Distance Dependency Components: Integrating the Focal-lesion and Neuroimaging Record

Maria Mercedes Piñango [1,*], Emily Finn [1,2], Cheryl Lacadie [2], and R. Todd Constable [2]

[1] *Language & Brain Lab, Department of Linguistics, Yale University, New Haven, CT, USA*
[2] *Magnetic Resonance Research Center, Yale University, New Haven, CT, USA*

Correspondence*:
Maria Mercedes Piñango
Department of Linguistics, 370 Temple St., New Haven, CT, 06511, United States of America, maria.pinango@yale.edu

SUPPLEMENTARY MATERIALS

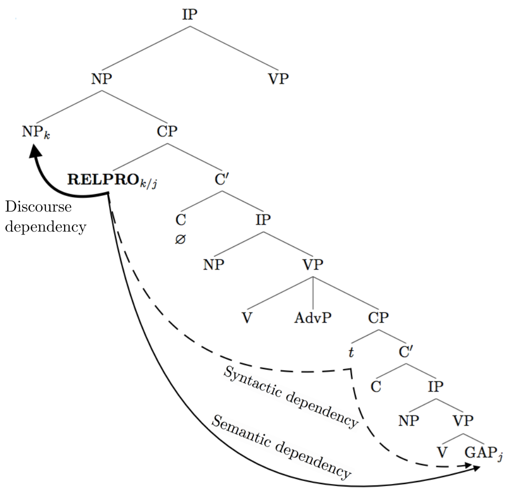

a. **Condition A**: The captain, **who** the sailor predicted yesterday **that** the weather would frighten$_{gap}$, turned back to port.

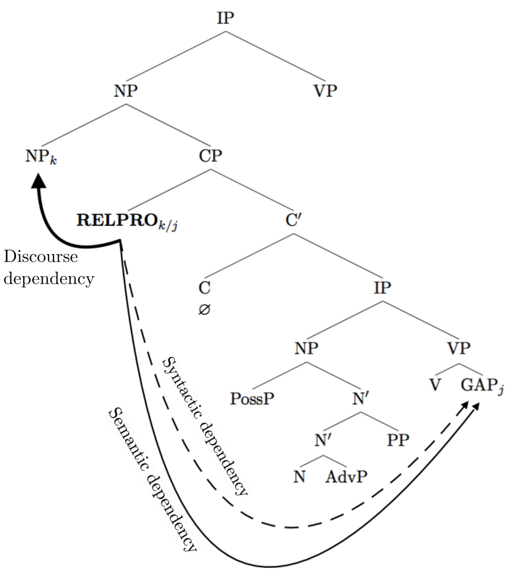

b. **Condition B**: The captain, **who** the sailor's prediction [yesterday about the weather] had frightened$_{gap}$, turned back to port.

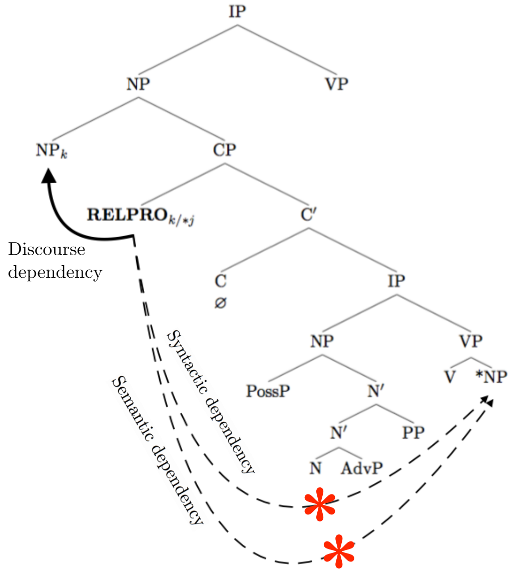

c. **Condition C**: *The captain, **who** the sailor's prediction yesterday about the weather had frightened **the crew**, turned back to port.

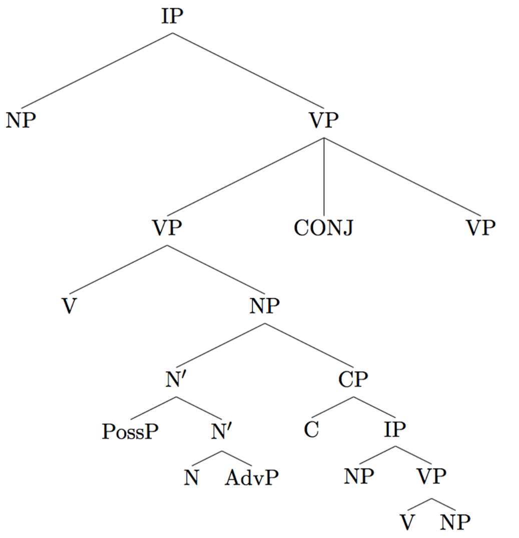

d. **Condition D**: The captain **believed** the sailor's prediction yesterday **that** the weather would frighten **the crew** and turned back to port.

Figure 1. Syntactic diagrams of experimental conditions indicating the respective LDDs.
